# *Drosophila* Activated Cdc42 Kinase Has an Anti-Apoptotic Function

**DOI:** 10.1371/journal.pgen.1002725

**Published:** 2012-05-17

**Authors:** Jessica A. Schoenherr, J. Michelle Drennan, Juan S. Martinez, Madhusudana Rao Chikka, Mark C. Hall, Henry C. Chang, James C. Clemens

**Affiliations:** 1 Department of Biochemistry, Purdue University, West Lafayette, Indiana, United States of America; 2 Department of Biology, Purdue University, West Lafayette, Indiana, United States of America; The Rockefeller University, United States of America

## Abstract

Activated Cdc42 kinases (Acks) are evolutionarily conserved non-receptor tyrosine kinases. Activating somatic mutations and increased ACK1 protein levels have been found in many types of human cancers and correlate with a poor prognosis. ACK1 is activated by epidermal growth factor (EGF) receptor signaling and functions to regulate EGF receptor turnover. ACK1 has additionally been found to propagate downstream signals through the phosphorylation of cancer relevant substrates. Using *Drosophila* as a model organism, we have determined that *Drosophila* Ack possesses potent anti-apoptotic activity that is dependent on Ack kinase activity and is further activated by EGF receptor/Ras signaling. Ack anti-apoptotic signaling does not function through enhancement of EGF stimulated MAP kinase signaling, suggesting that it must function through phosphorylation of some unknown effector. We isolated several putative *Drosophila* Ack interacting proteins, many being orthologs of previously identified human ACK1 interacting proteins. Two of these interacting proteins, Drk and yorkie, were found to influence Ack signaling. Drk is the *Drosophila* homolog of GRB2, which is required to couple ACK1 binding to receptor tyrosine kinases. Drk knockdown blocks Ack survival activity, suggesting that Ack localization is important for its pro-survival activity. Yorkie is a transcriptional co-activator that is downstream of the Salvador-Hippo-Warts pathway and promotes transcription of proliferative and anti-apoptotic genes. We find that yorkie and Ack synergistically interact to produce tissue overgrowth and that yorkie loss of function interferes with Ack anti-apoptotic signaling. Our results demonstrate how increased Ack signaling could contribute to cancer when coupled to proliferative signals.

## Author Summary

A number of recent studies have uncovered an involvement of Ack family members in human cancer. The majority of these studies focus on human ACK1 and suggest that ACK1 regulates diverse cancer-relevant biological functions, including stimulation of proliferation, blocking programmed cell death, and enhancing metastasis. It is unclear from these studies whether these biological outcomes are directly controlled by ACK1 activity or if they are indirect consequences of ACK1 signaling. Using *Drosophila* as a model organism, our study demonstrates that Ack serves to promote cell survival by blocking programmed cell death: a mechanism of eliminating excess, damaged, or cancerous cells. We further find that Ack activity functions synergistically with cell growth signals to produce massive cellular overgrowth. Our findings define the physiological role of Ack proteins and add further support to the value of Ack family members as therapeutic drug targets for the treatment of cancer.

## Introduction

**Figure 1:**
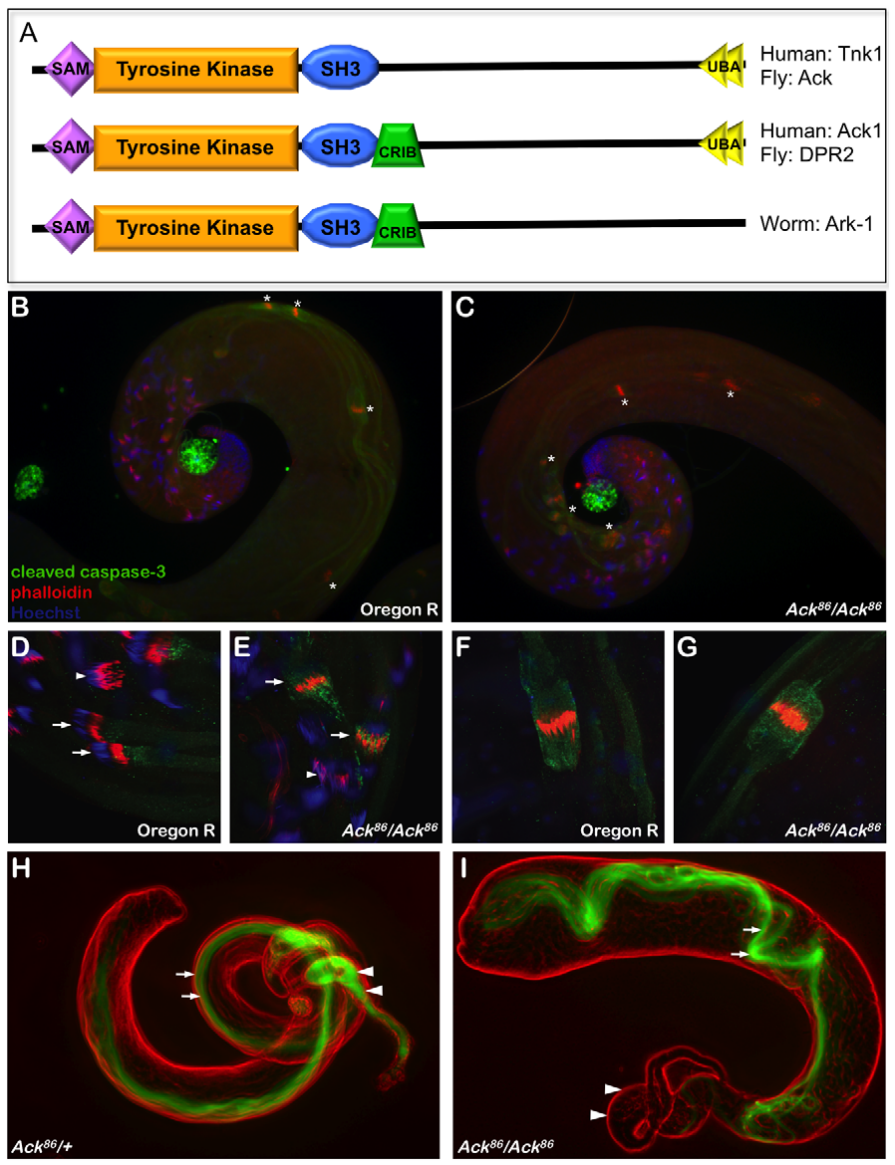
Ack is required for mature sperm production. (A) A schematic representation of Ack family members is shown. Ack family members can be grouped into three categories depending on their domain structure. All family members contain an N-terminal tyrosine kinase domain flanked by sterile alpha motif (SAM) and Src homology 3 (SH3) domains. The C-terminal half of these proteins also contains multiple proline-rich motifs. *Drosophila* Ack and human TNK1 do not contain a Cdc42/Rac interactive binding (CRIB) domain. All Ack family members except for Ark-1 in *C. elegans* contain an ubiquitin binding associated domain (UBA). (B–G) drICE activity (anti-cleaved caspase-3, green), actin rich investment cones (phalloidin, red) and DNA (DAPI, blue) are visualized in wild type (Oregon R) and Ack null mutant backgrounds (*Ack^86^/Ack^86^*) during sperm cell individualization. (B and C) The rostral or coiled region of the testis containing compacted nuclei is shown. The phalloidin staining individualization complexes (*) in wild type testis travel along the outer edge of the testis coil (B), while in Ack mutant testis they are positioned on the inside of the coil (C). (D–E) Higher magnification of individualization complexes assembling on condensed DNA (arrowheads) or beginning to migrate (arrows). (F–G) Higher magnification of more caudal migrating individualization complexes. (H–I) Visualization of dj-GFP fluorescence (green) shows the presence of elongated spermatids in the testes (arrows) and mature sperm in the seminal vesicle (arrowheads) of Ack^86^ hetrozygous males (H). (I) Elongated spermatids are present in the testes of Ack^86^ homozygous males (arrows) but no mature sperm are present in the seminal vesicle (arrowheads).

Activated Cdc42 kinases (Acks) are non-receptor tyrosine kinases that are evolutionarily conserved. The founding member of this family is human ACK1, which was identified as a protein that binds to CDC42 in its active GTP bound form [1]. Since this discovery Ack homologs have been found in chordates, arthropods and nematodes. Ack family members can be divided into three structural categories based on the presence or absence of four conserved domain motifs (Figure 1A). All Ack family members contain an amino-terminal tyrosine kinase domain that is flanked by a sterile alpha motif (SAM) and a Src homology 3 (SH3) domain. The carboxy-terminal half of these kinases contains short proline rich sequences, but lacks any identifiable domains, with the exception of two tandemly repeated ubiquitin-associated (UBA) domains located at the extreme carboxy-terminus [2]–[4]. ACK1 UBA domains have been shown to interact with both mono and poly-ubiquitinated proteins [5]–[7] and are thought to play a role in ACK1 protein turnover [6]. The *Caenorhabditis elegans* Ack homolog, Ark-1, contains no UBA domains, placing it in a class by itself. The other two Ack structural classes can be distinguished by the presence or absence of a Cdc42/Rac interactive binding (CRIB) domain. Human ACK1 and *Drosophila* PR2 are representative members of the CRIB domain containing structural class, while human TNK1 and *Drosophila* Ack are members of the structural class lacking a conserved CRIB domain. Variants containing a CRIB domain bind GTP liganded CDC42, but this interaction does not appear to directly influence Ack activity *in vitro* [2].

Human ACK1 is the most well characterized member of the Ack family. Early studies uncovered a role for ACK1 in the promotion of internalization and down-regulation of activated epidermal growth factor (EGF) receptor. ACK1 tyrosine phosphorylation is enhanced and ACK1 is co-localized with EGF receptor after EGF stimulation [8], [9]. Knockdown of ACK1 reduces the rate of EGF receptor degradation following EGF stimulation [5]. While on the surface these data suggest that ACK1 merely serves as a negative regulator of growth factor signaling, ACK1 activation may additionally propagate downstream signaling. Recent studies support this latter alternative by uncovering a role for ACK1 as a positive transducer of cell surface receptor signaling that promotes growth and survival by ACK1 mediated phosphorylation and activation of downstream components, including AKT [10] and the androgen receptor [11].

A pro-survival role for Ack function is consistent with reported links between activation of Ack family members and cancer genesis and metastasis. Several somatic missense mutations have been identified in ACK1 from cancer tissue samples that increase ACK1 autophosphorylation and promote cellular proliferation and migration [12]. Amplification of the ACK1 gene in tumors correlates with a poor prognosis, and ACK1 overexpression in cancer cell lines increases invasiveness in a mouse metastasis model [13], while knockdown of ACK1 reduces the migration of human breast cancer cells [14], [15].

Activated ACK1 has been detected in advanced human prostate cancers [13], [16] where it has been shown to phosphorylate three cancer relevant substrates in prostate cancer cell lines: WWOX [16], AKT [10], and androgen receptor [11]. *WWOX* spans the *FRA16D* chromosomal fragile site that is frequently disrupted in human cancers [17]–[19]. While the molecular function of WWOX is not known, it has been shown that the growth of tumor cells lacking WWOX is strongly inhibited by restoring WWOX expression [20]. ACK1 phosphorylation of WWOX leads to the polyubiquitination and degradation of WWOX, which correlates with a tumorigenic role [16]. AKT is a serine/threonine kinase whose activity promotes cell survival and proliferation, while deregulation of the AKT signaling pathway is commonly associated with cancer [21]. ACK1 activation results in tyrosine phosphorylation and apparent activation of AKT in a PI3K independent mechanism [10]. Finally, the activity of the androgen receptor is required for growth of prostate cells. In advanced stages of prostate cancer, these cells lose their dependence on androgens for activation of this receptor to become androgen independent prostate cancer [22], [23]. ACK1 has been found to phosphorylate the androgen receptor, promote androgen independent growth of prostate cells, and activate transcription of androgen inducible genes in the absence of androgen [11].

Less is known about the function of Ack family members lacking CRIB domains, and published studies on TNK1 describe conflicting functions. TNK1 overexpression in cell culture lines inhibits cell growth in a kinase dependent manner [24]. Mutant mice having deletions in the kinase domain of TNK1 develop spontaneous tumors at a high frequency, which is thought to originate from hyperactivation of Ras signaling [25], [26] and suggests that TNK1 functions as a tumor suppressor. In contrast to this function, TNK1 was identified as a potentially oncogenic tyrosine kinase in a mutagenesis screen [27] and activated TNK1 was found in Hodgkin's lymphoma [28]. It is possible that these conflicting findings reflect tissue specific responses or complex dosage sensitivity to TNK1 loss and gain of function.

In order to better understand the physiological role of Ack family members and determine how Ack might contribute to cancer, we conducted genetic and biochemical experiments in the model organism *Drosophila melanogaster*. Our studies focus on *Drosophila* Ack, which has a domain structure resembling human TNK1 (Figure 1A), but shares significantly higher sequence identity with ACK1 in all conserved domains including the kinase domain activation loop. We find that *Drosophila* Ack possesses potent anti-apoptotic properties that function downstream of EGF receptor signaling through an unknown mechanism. This activity is dependent on Ack kinase function and can be further stimulated by increased Ras signaling. We have conducted a protein interaction study and find that many of the same proteins that associate with human ACK1 also bind to fly Ack. The influence of these proteins on Ack anti-apoptotic activity was tested, and we determined that the adapter protein Drk (GRB2) is required for this activity, while the transcriptional co-activator protein yki (YAP) functions synergistically with Ack to promote cell survival and massive tissue overgrowth. Our findings support both anti-apoptotic and proliferative roles for Ack family members, which may contribute to cancer genesis and progression.

## Results

Loss-of-function alleles for both *Drosophila* Ack and PR2 have been described previously [29]. *Ack^86^* is a protein null allele that was generated by imprecise excision of the P element insertion KG00869 [29], [30]. Homozygous *Ack^86^* flies appear visually normal, although females homozygous for *Ack^86^* are fertile while homozygous males are sterile [29]. Genes that are involved in programmed cell death regulation and execution are implicated in Drosophila male fertility because apoptosis-like events are required for sperm cell individualization [31]. Given that Ack null males are sterile and that Ack is implicated in human cancer, we reasoned that Ack might function to regulate apoptosis. Analysis of one day old adult testis reveals disorganized patterns of elongated individualizing spermatids in *Ack^86^* homozygous animals compared to wildtype controls (Figure 1B–1G). The individualization complexes are displaced from the outer region of the testis coil to the inner region in Ack mutants (Figure 1B and 1C). Cleaved caspase-3 staining is also more intense surrounding and associated with the investment cone structures in newly migrating individualization complexes in Ack mutants compared to controls (Figure 1D and 1E). Once the individualization complexes have migrated caudally, the levels of cleaved caspase-3 immunoreactivity are comparable in both Ack mutants and controls (Figure 1F and 1G). dj-GFP (don juan), which decorates the elongated mitochondria, was used to visualize spermatids in the testes and mature sperm in the seminal vesicles [32], [33]. Both Ack^86^ heterozygous and homozygous testes contained dj-GFP-positive spermatids (arrows, Figure 1H and 1I). However, while dj-GFP-positive sperm were present in heterozygous seminal vesicles (arrowheads, Figure 1H), Ack^86^ null seminal vesicles were empty (arrowheads, Figure 1I), indicating that Ack has a role in mature sperm production.

### Ack Suppresses Cell Death Induced by Hid

**Figure 2:**
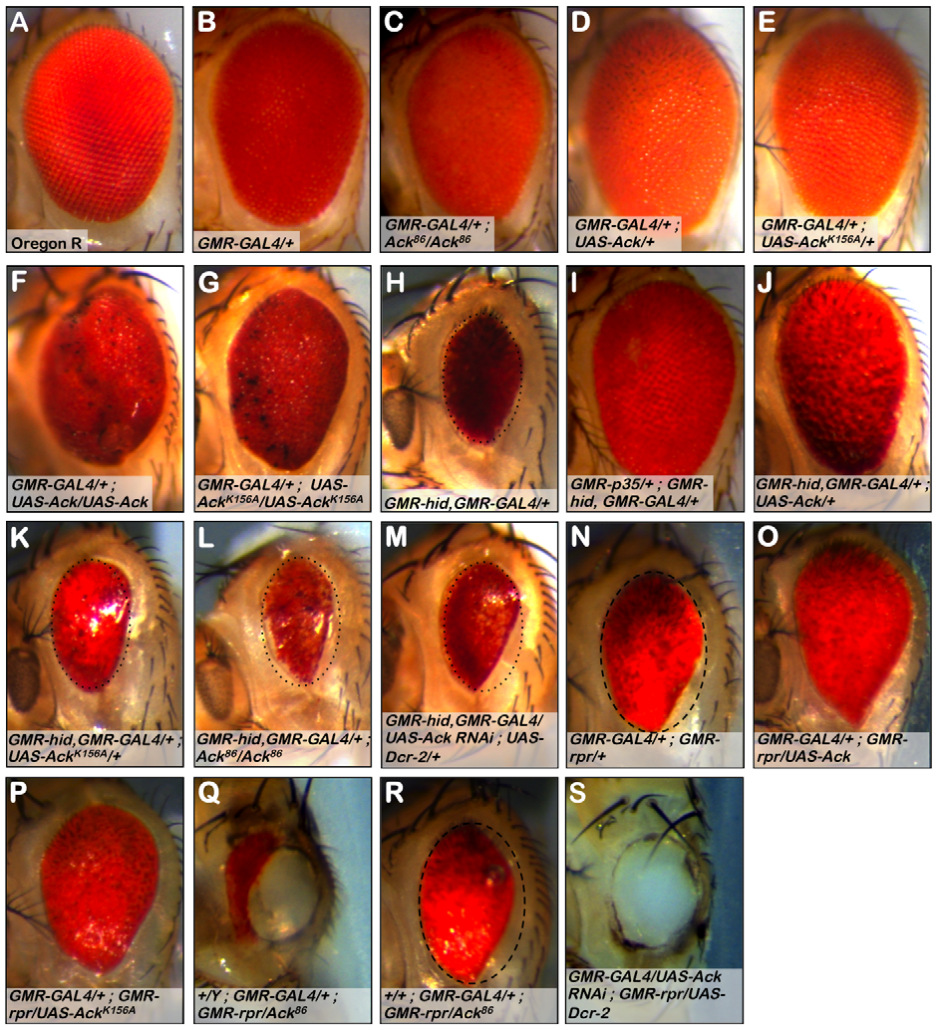
Ack suppresses small eye phenotypes induced by hid and rpr. The *Drosophila* eye consists of a hexagonal array of roughly 800 simple eyes called ommatidia. The effect of loss-of-function or expression of Ack family member transgenes on ommatidia patterning and apoptosis is assessed in a *GMR-GAL4* background (B–G), the apoptosis induced backgrounds containing *GMR-hid*, *GMR-GAL4* (H–M) or *GMR-GAL4*, *GMR-rpr* (N–S). Genotypes are indicated in each panel. (A) Oregon R wild type eye. (B) *GMR-GAL4* induces weak disorganization of ommatidia patterning. All eyes containing *GMR-Gal4* exhibit this roughness. (C) Ack protein loss (null) produces a slightly smaller eye. (D) Overexpression of Ack results in a slightly increased eye size. (E) Overexpression of kinase inactive Ack^K156A^ is comparable to the Ack null eye in panel C. (F and G) Expression from two copies of Ack (F) or Ack^K156A^ (G) transgenes results in a highly disorganized eye. (H) The death-inducing stimulus hid induces programmed cell death (PCD) resulting in a small eye phenotype, which serves as the basis of comparison for panels I–M. To aid in eye size comparison we included a dotted oval that has been reproduced in panels K–M. (I) Expression of the caspase inhibitor p35 blocks PCD and results in an eye size similar to Oregon R. (J) Ack overexpression suppresses the hid small eye phenotype. (K) Kinase inactive Ack^K156A^ is unable to suppress the hid induced small eye phenotype. (L and M) Ack loss of function (L) and Ack knockdown with RNAi (M) enhances the hid small eye phenotype. (N) The death-inducing stimulus rpr also induces PCD and results in a small eye phenotype, which serves as the basis of comparison for panels O–S. (O and P) Overexpression of Ack (O) or Ack^K156A^ (P) suppresses the small eye phenotype induced by rpr expression. (Q) Dosage reduction of Ack in an rpr expressing background results in partial ablation of the eye, leaving a hole in the head exoskeleton in all males and the majority of females with this genotype. (R) Female escapers having single copy dosage reduction in a rpr expressing background have eye sizes slightly reduced compared to rpr/GAL4 eyes. The dashed oval in panel N is reproduced in panel R to aid in size comparison. (S) Further reduction of Ack by RNAi mediated knockdown results in loss of all eye tissue including lens cuticle resulting in a hole in the head exoskeleton of pharate adults. White tissue seen in cuticle holes is brain optic lobe (Q and S).

The *Drosophila* compound eye has been used as an experimental system to assess apoptotic gene function and dissect apoptotic signaling pathways [34], [35]. Ack null flies have eyes that appear normal as do flies that overexpress Ack or kinase inactive Ack (Ack^K156A^) using a promoter that drives expression in the developing eye disc (*GMR-Gal4*, *UAS-Ack* or *Ack^K156A^*, Figure 2A–2E). A previous study reported that eye expression of either Ack or a kinase dead variant Ack^K156A^ using *GMR-Gal4* resulted in massive disorganization of the eye [4]. We find that we can replicate these phenotypes by doubling the dose of Ack or Ack^K156A^ through the introduction of a second copy of the Ack transgene (Figure 2F and 2G), indicating that our Ack transgenes express proteins at comparatively lower levels. Therefore, any eye size changes seen can be attributed to a genetic interaction with Ack and not due to Ack overexpression itself.

*Reaper (rpr), head involution defective* (*hid*) and *grim* [34], [36], [37] are three cell death genes that function as antagonists of *Drosophila* inhibitor of apoptosis (DIAP) [38]. The BIR domain of DIAP binds to initiator and effector caspases to block their proteolytic activity [39]. IAP antagonists competitively bind to the BIR domain to dissociate bound caspases [40], leading to caspase activation and apoptotic cell death. Expression of hid using *GMR-GAL4* results in a reduced eye size (Figure 2H). Co-expression of the baculovirus caspase inhibitor p35 with hid completely suppresses the small eye phenotype (Figure 2I), demonstrating that the reduction of eye size caused by hid expression is due to the activation of caspases, resulting in apoptosis of cells that make up the eye during development.

To determine if Ack contributes to the regulation of cell survival, we assessed the ability of various Ack transgenes and alleles to modify the small eye phenotype induced by hid expression. Co-expression of Ack with hid greatly suppresses the hid induced small eye phenotype, demonstrating that Ack may function in a survival role (Figure 2J). Kinase inactive Ack^K156A^ expression fails to modify the hid small eye phenotype, indicating that Ack kinase activity is required for inhibition of hid induced eye size reduction (Figure 2K). When Ack function is removed, either genetically or by RNAi, enhancement of the hid small eye size is observed (Figure 2L and 2M).

Additionally, we assessed the ability of Ack to suppress the small eye phenotype induced by rpr expression in the eye. Expression of wildtype Ack or kinase inactive Ack^K156A^ suppresses the small eye phenotype induced by rpr expression (Figure 2N–2P), suggesting that non-kinase functions of Ack may be important for suppressing rpr function. Single copy loss or RNAi mediated knockdown of *Ack* expression in rpr expressing fly eyes results in flies that fail to eclose. Dissection of the pharate adults contained within the pupa cases revealed stunning phenotypes. Gene dosage reduction of *Ack* in a *GMR-rpr* background results in eyes that are partially ablated (Figure 2Q). A few female escapers eclosed (no male escapers were found) that had eye sizes slightly smaller than *GMR-GAL4*; *GMR- rpr* (Figure 2R). Further reduction of *Ack* dosage by RNAi in the *GMR-rpr* background results in complete ablation of the eye (Figure 2S) with no escapers. In both cases, the ablated eye area is replaced by a hole in the head cuticle where eye tissue would normally reside (Figure 2Q and 2S). Further dissection of the heads of these animals determined that the white tissue visible in place of the eye is not actually eye tissue, but is instead the optic lobe region of the *Drosophila* brain, which in some animals protrudes outside of the head (as in Figure 2S). Given the shape and location of the holes, we hypothesize that the eye developed to a point and then was partially or completely consumed by massive activation of apoptosis prior to deposition of lens cuticle during pupa development. We conclude from these experiments that Ack has anti-apoptotic or proliferative properties capable of overcoming the effects of hid and rpr induced apoptosis.

Finally, overexpression of PR2 or knockdown of PR2 using RNAi fails to significantly modify the hid or rpr induced small eye phenotypes (Figure S1). We conclude that unlike Ack, PR2 is either not involved in survival regulation or may require additional factors, such as interaction with Cdc42-GTP, for activation of its kinase activity.

### Ack Suppresses Apoptosis

**Figure 3:**
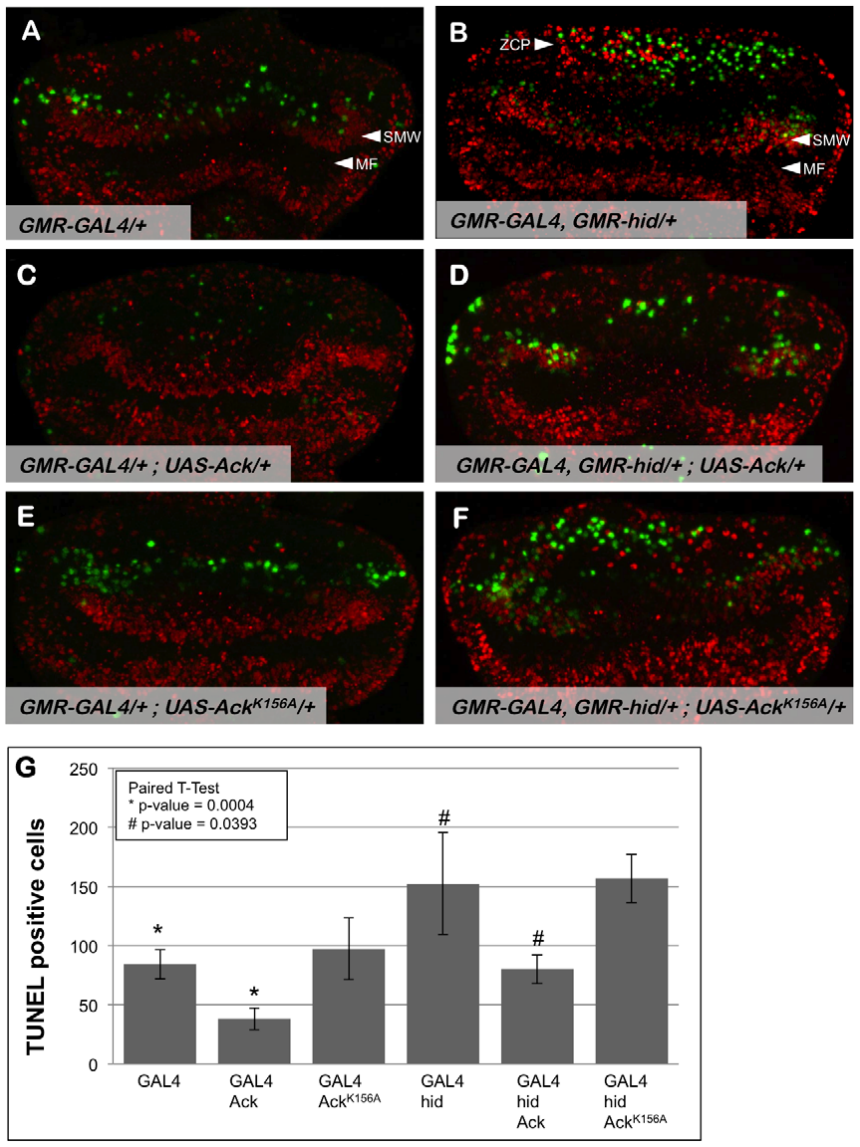
Ack expression produces fewer TUNEL positive cells. Proliferation and apoptosis in *Drosophila* third instar eye discs (posterior is up) were analyzed by BrdU incorporation (red) and TUNEL (green), respectively. Genotypes are indicated in each panel; the morphogenetic furrow (MF) is labeled in panels A and B. (A) *GMR-Gal4* eye discs have TUNEL positive cells posterior to the second mitotic wave (SMW). (B) Hid expression results in TUNEL staining posterior to the SMW as well as in the posterior region of the eye disc within the zone of compensatory proliferation (ZCP). (C) Ack overexpression results in fewer TUNEL positive cells and no observable change in BrdU incorporation patterns. (D) TUNEL staining is also decreased when Ack is expressed in a hid background. The Ack mediated decrease in apoptosis also decreases the number of BrdU positive cells in the location of the ZCP as expected. (E and F) Ack kinase dead mutant shows an enhancement of cell death post furrow. (G) Quantification of TUNEL assay from 4 different eye discs per genotype. P-values using a paired t-test verifies that Ack expression is statistically significant compared to control (* = 0.004, # = 0.0393), whereas, Ack kinase inactive mutant is not statistically significant (* = 0.8007, # = 0.765)(GraphPad Software, QuickCalcs online).

Ack mediated modification of the hid induced small eye phenotype could be achieved through two mechanisms. Ack could function in an anti-apoptotic role to block pro-apoptotic events initiated by overexpression of hid or rpr. Alternatively, Ack activity could stimulate cellular proliferation such that apoptotic cells are replaced by additional cell division of surviving cells. Again, the *Drosophila* eye serves as an ideal system to distinguish between these possibilities. Patterning of the *Drosophila* eye occurs during the third instar larval stage. During pattern formation, a wave of differentiation known as the morphogenetic furrow (MF) moves anteriorly across the eye imaginal disc [41]. Cells divide asynchronously ahead of the furrow; however, cell division following the furrow is tightly regulated and only occurs once more in a synchronous band that follows behind the furrow known as the second mitotic wave (SMW) [42]. The GMR synthetic promoter drives expression of proteins in cells behind the morphogenetic furrow. Expression of hid via the GMR promoter has been shown to increase apoptotic cells posterior to the furrow, but it also induces an additional region of cell division called the zone of compensatory proliferation (ZCP) that is posterior to the SMW (Figure 3B) [43], [44].

Use of the GMR synthetic promoter to express hid and Ack transgenes allows us to assess whether Ack expression leads to inhibition of apoptosis or stimulation of proliferation by TUNEL staining and BrdU incorporation, respectively, in the third instar eye disc. Expression of hid in the eye disc produces an increase in TUNEL positive cells in the region of the SMW and in the most posterior region of the disc containing the ZCP (compare Figure 3A and 3B). Expression of Ack in the eye disc substantially reduces the number of TUNEL positive cells in both regions (Figure 3C and 3D), while expression of kinase inactive Ack^K156A^ appears to increase the number of TUNEL positive cells (Figure 3E and 3F). Ack or Ack^K156A^ expression does not appear to affect the number of BrdU positive cells. These data demonstrate that Ack kinase activity functions in an anti-apoptotic manner (see Figure 3G for quantification).

It is possible that Ack specifically functions within the eye to modulate programmed cell death activation. To address this we adapted a system that had been developed to study regenerative growth in imaginal discs [45]. In this system *UAS-rpr* is driven by *rotund* (*rn*)*-GAL4*, which is active in several imaginal discs including the wing disc. Reaper expression in this system is further modulated due to the inclusion of a *GAL80^ts^* transgene, which allows for the temporal activation of UAS controlled transgenes by raising the temperature from 18°C to 30°C. We determined that a three hour exposure to 30°C followed by a four hour recovery at 18°C was sufficient to produce an intermediate amount of programmed cell death in the wing disc pouch as assessed by TUNEL (Figure S2A). Expression of Ack in this background decreased the number of TUNEL positive cells in the wing disc (Figure S2B) while expression of kinase inactive Ack^K156A^ or dosage reduction of Ack using the *Ack^86^* allele increased TUNEL positive cells (Figure S2C and S2D). We conclude that Ack anti-apoptotic function is not limited to the *Drosophila* eye.

### The Anti-Apoptotic Properties of Ack Function Independently of MAP Kinase Signaling

**Figure 4:**
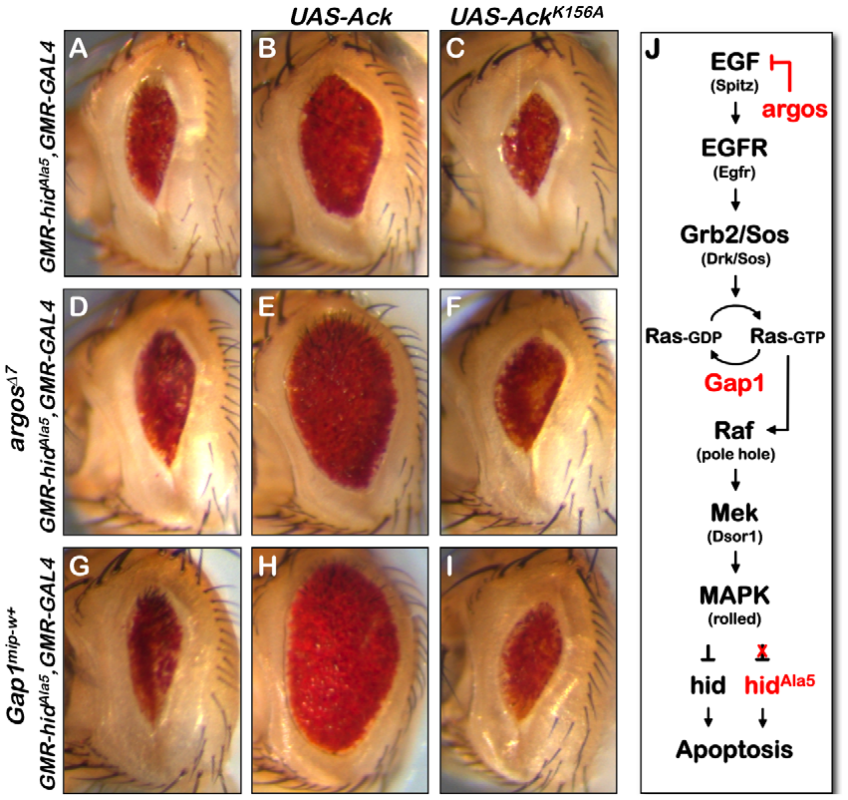
Ack anti-apoptotic function is stimulated by EGF receptor/Ras signaling. The *Drosophila* eye size assay is used to determine how Ack functions with respect to EGF receptor/MAPK signaling. (A) Hid^Ala5^ control eye. (B) Overexpression of Ack results in suppression of PCD induced by hid^Ala5^ indicating that Ack suppression of apoptosis is independent of hid phosphorylation by MAPK. (C) Kinase inactive Ack fails to suppress PCD, showing that kinase activity is required for Ack suppression of hid^Ala5^ induced PCD. (D and G) Increased signaling through the MAPK pathway by single copy loss of *argos* (D) or *Gap1* (G) fails to suppress apoptosis due to the inability of MAPK to phosphorylate and inhibit hid^Ala5^. (E and H) Ack overexpression combined with single copy loss of *argos* (E) or *Gap1* (H) further suppresses hid^Ala5^ compared to panel B, showing that Ack can be activated by EGF receptor or Ras signaling. (F and I) Ack^K156A^ combined with single copy loss of *argos* (F) or *Gap1* (I) does not suppress hid^Ala5^ PCD. (J) A schematic of the EGF initiated signaling pathway through MAPK is shown. The MAPK pathway regulates hid induced PCD by phosphorylating and inhibiting hid. A hid mutant having five serine/threonine phosphorylation sites mutated to alanine (hid^Ala5^) is unable to be inhibited by the MAPK pathway.

EGF receptor signaling regulates promotion of apoptosis by hid expression in the *Drosophila* eye [35], [46]. Specifically, EGF receptor activation leads to the activation of mitogen activated protein kinase (MAPK), via the Ras/Raf/MEK pathway. In *Drosophila*, activated MAPK directly phosphorylates hid on multiple serine and threonine residues, which inactivates the pro-apoptotic function of hid [35]. A schematic representation of this pathway is shown (Figure 4J). Expression of hid^Ala5^, a mutant in which the serine or threonine of 5 putative MAPK sites have been changed to alanine, induces apoptosis which can not be suppressed by MAPK activity [35].

Vertebrate Ack studies have implicated ACK1 in the turnover and regulation of EGF receptor signaling [5], [7], [47]. Therefore, it is possible that Ack may influence apoptosis by modulating EGF receptor activity itself or downstream components that ultimately stimulate MAPK. In order to test this possibility, we assessed the ability of Ack expression to block programmed cell death stimulated by hid^Ala5^ expression in the *Drosophila* eye. Expression of hid^Ala5^ using the GMR promoter produces a reduced eye size due to the induction of apoptosis (Figure 4A). The eye size reduction caused by hid^Ala5^ is greater than that seen by expression of hid (compare Figure 4A and 2H). This variation could simply be due to differences in expression levels of the hid transgenes caused by genomic insertion site positional effects. Alternatively the differences could be explained by the activity of endogenous MAPK, which is able to partially block apoptosis induced by hid, but not by hid^Ala5^.

We selected two proteins that are known to inhibit EGF receptor signaling or Ras/Raf/MEK signaling. Argos inhibits EGF receptor activation by competitively binding extra-cellular EGF, while Gap1 attenuates Ras signaling by facilitating the conversion of Ras-GTP to the inactive Ras-GDP form [48], [49]. Reducing the protein levels of these inhibitors increases signaling through the EGF receptor pathway, resulting in increased MAPK activation. Reduction of protein levels can be achieved by reducing gene dosage through introduction of a single copy of a strong hypomorphic allele. Single copy loss of either *argos* or *Gap1* has been found to block hid induced apoptosis [35]. However, single copy loss of *argos* or *Gap1* is unable to block apoptosis induced by hid^Ala5^ expression and fails to suppress the small eye size (Figure 4D and 4G and [35]).

If Ack kinase activity blocks apoptosis by enhancing signaling through the Ras/Raf/MEK pathway, then we would expect that Ack expression would be unable to suppress apoptosis induced by hid^Ala5^ expression. However, we find that expression of Ack is able to suppress the small eye that is induced by both hid and hid^Ala5^ (compare Figure 2H to 2J and Figure 4A to 4B). In both cases, Ack expression induces a similar fold increase in eye size: roughly a 1.8 fold increase in eye area for hid and a 1.6 fold increase for hid^Ala5^. Taken together, our data demonstrate that Ack kinase activity blocks programmed cell death induced by hid through a MAPK independent mechanism.

### Ack Anti-Apoptotic Activity Is Activated by EGF/Ras Signaling

In vertebrates, ACK1 has been shown to be tyrosine phosphorylated and localized to the EGF receptor upon EGF stimulation [5], [50], [51]. This is largely thought to lead to the internalization and degradation of the EGF receptor. Our findings raise the possibility that in addition to EGF receptor turnover, Ack activation may signal to uncharacterized downstream components to regulate programmed cell death. By using hid^Ala5^ to induce programmed cell death, we eliminate the pro-survival signaling of the EGF receptor through MAPK. If EGF signaling activates Ack, then we would expect that the introduction of mutations that activate the EGF receptor or downstream components would result in an increase in the ability of Ack to suppress apoptosis. This is in fact what we observe: gene dosage reduction of *argos* or *Gap1* combined with expression of Ack further suppresses the hid^Ala5^ induced small eye phenotype (compare Figure 4B to 4E and 4H). These data suggest that Ack may not be directly activated by the EGF receptor, but may instead be activated at the level of Ras-GTP or further down the Raf/Mek/MAPK pathway. Finally, suppression of apoptosis is absolutely dependent on the kinase activity of Ack, as Ack^K156A^ shows no suppression of the hid^Ala5^ small eye phenotype either alone or in conjunction with mutations that enhance EGF receptor or Ras signaling (Figure 4C, 4F and 4I).

**Figure 5:**
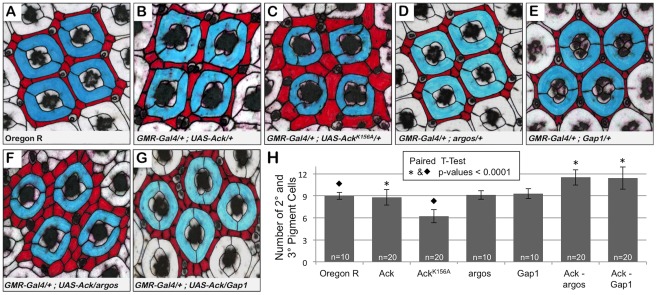
Ack expression genetically interacts with *argos* and *Gap1* loss of function. Genotypes are shown in each panel. (A–G) 42% pupa retina dissections stained with anti-armadillo and false colored to indicate primary (blue) and secondary or tertiary (red) pigment cells. (A) Oregon R, wildtype phenotype contains nine interommatidial pigment cells and three bristle cells per ommatidial unit. (B) Overexpression of Ack produces a duplication of bristles and slight disorganization; however, there is still an average of 9 pigment cells present surrounding each core unit. (C) Expression of kinase inactive Ack results in fewer but larger pigment cells. (D–E) *Gap1* single copy loss (D) and *argos* single copy loss (E) do not affect eye patterning. (F–G) Single copy loss of *argos* (F) or *Gap1* (G) in combination with Ack overexpression results in increased interommatidial pigment cells. (H) Quantification of pigment cell number from all dissections. P-values using a paired t-test verify that the increased number of pigment cells when Ack is combined with modifiers of the EGF receptor pathway is statistically significant compared to control (* = 0.0001). Loss of pigment cells in the Ack kinase inactive mutant is also statistically significant compared to the Oregon R control (⧫ = 0.0001)(GraphPad Software, QuickCalcs online).

Next we sought to determine if Ack activity is able to block programmed cell death that occurs during normal developmental tissue patterning. The *Drosophila* compound eye is composed of 800 simple eye units called ommatidia comprising eight photoreceptors, four cone cells and two primary pigment cells. Each ommatidium is surrounded by a regular hexagonal array of 12 interommatidial cells comprising six secondary pigment cells, three tertiary pigment cells and three bristles (Figure 5A). The lattice of interommatidia cells starts to form at 20% pupal development and is completed by 33% of pupal development. During this time period, excess interommatidial cells are selectively eliminated by programmed cell death. Ack expression results in an increase in the number of bristles without significantly affecting the number of pigment cells as assessed at 42% pupa development (Figure 5B and 5H). Expression of Ack^K156A^ also increases the number of bristles within the interommatidia lattice, but results in a 33% decrease in pigment cell number (Figure 5C and 5H). Single copy loss of *argos* or *Gap1* does not affect bristle or pigment cell number (Figure 5D, 5E and 5H) on their own, but produces a 25% increase in pigment cell number when combined with Ack expression (Figure 5F–5H). These data further support an anti-apoptotic function for Ack that can be activated by EGF/Ras signaling and suggest that kinase inactive Ack^K156A^ expression enhances programmed cell death activation in a background not containing overexpression of DIAP antagonists.

### Identification of Ack Interacting Proteins

Our results indicate that Ack kinase activity is anti-apoptotic and that this activity can be enhanced by activation of the EGF receptor or its downstream signaling components such as Ras. The proteins that link EGF/Ras signaling to *Drosophila* Ack activation and the downstream targets of Ack are not known. Several vertebrate ACK1 interacting proteins have been identified (see [52] for a comprehensive list) and some of these (including AKT, GRB2, NCK, NEDD4 and WWOX) are attractive candidates for regulation or transduction of Ack anti-apoptotic signaling.

**Figure 6:**
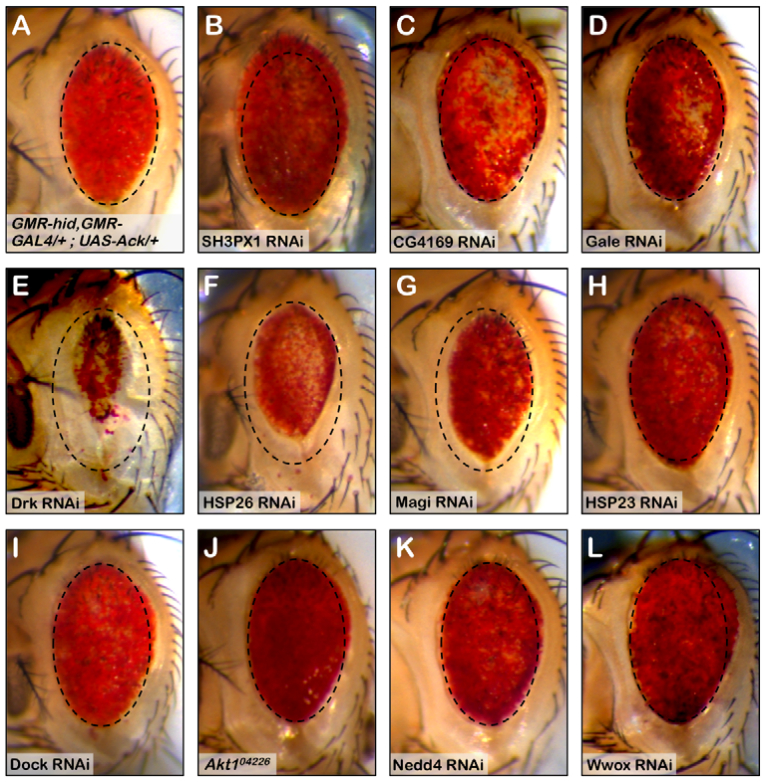
RNAi knockdown of Ack interacting proteins identify Drk as part of the Ack survival pathway. The involvement of Ack physically interacting proteins identified in our studies and elsewhere is tested in the *Drosophila* eye size assay by RNAi mediated knockdown. (A) Hid^Ala5^, Ack overexpression baseline eye. To aid in eye size comparison we included a dashed oval that has been reproduced in all panels. (B–I and K–L) RNAi transgenes are tested in the *GMR-hid^Ala5^*, *GMR-Gal4/+*; *UAS-Ack/+* background containing a *UAS-Dcr-2* transgene on chromosome II or III depending on *UAS-RNAi* transgene location. The *UAS-RNAi* transgene target is indicated in each panel. (J) *GMR-hid^Ala5^*, *GMR-Gal4/+*; *UAS-Ack/Akt1^04226^* (Akt1 loss of function allele). (B, C and L) SH3PX1, CG4169 and Wwox RNAi result in further suppression of PCD. (E–G) Drk, HSP23 and Magi RNAi result in enhancement of PCD. (D, and H–K) The other candidates do not modify the hid^Ala5^ Ack baseline and thus are most likely not a part of the Ack anti-apoptotic pathway.

We utilized a biochemical approach to identify Ack interacting proteins from *Drosophila* Schneider 2 (S2) cells (see Materials and Methods for details). We identified several Ack interacting proteins that are fly orthologs of known vertebrate ACK1 interacting proteins, including Clathrin, Drk (GRB2), Hsp83 (HSP90), Dock (NCK), WASp (WAS) and SH3PX1 (SNX9). The top ten hits based on Mascot (probability) score that specifically co-purify with Ack and are absent from control preparations are shown (Table 1). RNAi lines for each of the proteins in Table 1 as well as Dock, Akt1, Nedd4 and Wwox were obtained and used to determine if knockdown of these proteins influences the ability of Ack to rescue hid^Ala5^ induced programmed cell death. Weak or no modification of eye size was seen for the candidate Ack interacting proteins CG4169, Gale, Hsp23 and Dock. The ACK1 interacting proteins not identified in our dataset (Akt1, Nedd4 and Wwox) also failed to robustly modify the eye size in this background (see Figure 6). Additionally, we obtained loss and gain of function alleles for Akt1, but could detect only minor enhancement or suppression, respectively, of hid or hid^Ala5^ induced programmed cell death, which appeared to produce an additive effect when combined with Ack expression (Figure S3).

**Table 1:** Top ten Ack interacting proteins based on their Mascot score.

Protein	HGNC Ortholog name	Unique peptide number	Mascot score^a^	Molecular Function	Effect of knockdown on Ack anti-apoptotic activity
SH3PX1	SNX9/18/33	21	930	Phosphoinositide binding	enhancement
Yorkie	YAP1	9	612	Transcriptional coactivation	lethal
HSP 83	HSP90AA1	9	375	Molecular chaperone	lethal
CG4169	UQCRC2	9	281	Ubiquinol-cytochrome-c reductase core protein 2	weak enhancement
Gale	GALE	6	272	UDP-galactose 4′-epimerase	no effect
Drk	GRB2	5	259	SH2/SH3 adaptor protein	strong suppression
HSP 26	HSPB1	7	259	Molecular chaperone	suppression
Pgam5	PGAM5	3	238	Phosphatase	no effect
Magi	MAGI1	4	226	Guanylate kinase activity	weak suppression
HSP 23	HSPB1	8	215	Molecular chaperone	no effect

a Mascot score = −10log(P) where P is the probability that the match is incorrect.

SH3PX1 RNAi suppresses the hid^Ala5^ induced small eye phenotype in the Ack expressing background, while Hsp26 and Magi RNAi enhance the small eye phenotype. These data indicate that SH3PX1, Hsp26 and Magi may function directly with Ack or in a parallel pathway to regulate cell survival. Hsp83, Yorkie and Drk had the largest effect and were further analyzed: RNAi knockdown of Hsp83 and Yorkie in a hid^Ala5^ and Ack expressing background resulted in flies that failed to eclose from pupa cases, while Drk knockdown produced adults that had greatly reduced eye size (Figure 6E). Based on our results, we elected to further characterize the requirement of Drk and yki for Ack anti-apoptotic function.

### Drk Is Required for Ack Anti-Apoptotic Function

**Figure 7:**
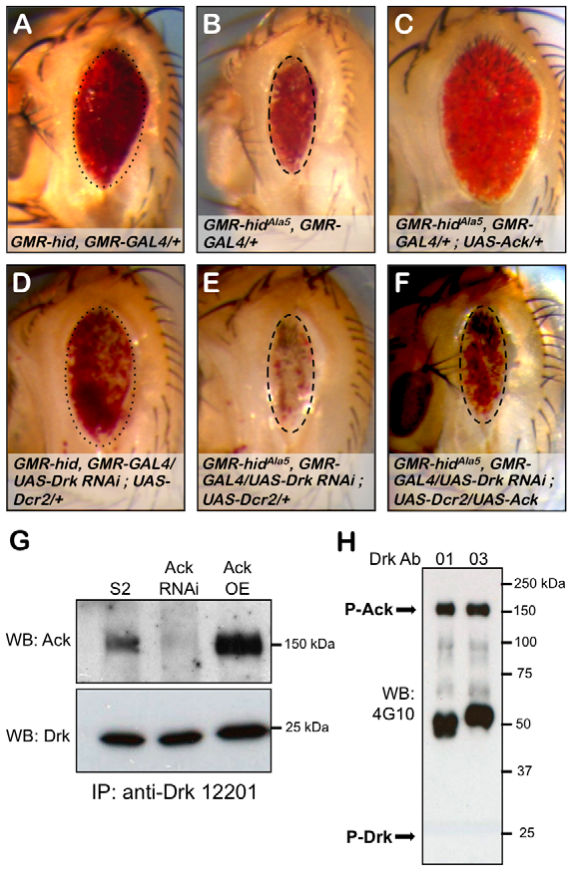
Drk is required for Ack anti-apoptotic activity. The influence of Drk loss of function on PCD was assessed using the eye size assay. Genotypes are indicated in each panel. Hid (A) and hid^Ala5^ (B) backgrounds were combined with RNAi mediated knockdown of Drk (D and E). Hid and hid^Ala5^ unmodified eye sizes have been outlined with dotted and dashed ovals that have been reproduced in panels D and E respectively. Drk knockdown in the hid (D) or hid^Ala5^ (E) backgrounds results in no reduction in eye size but a loss of pigmentation, which is more pronounced with hid^Ala5^. (C and F) The requirement of Drk for Ack anti-apoptotic function is assessed. (C) Hid^Ala5^, Ack overexpression baseline eye. (F) RNAi mediated knockdown of Drk inhibits Ack's ability to suppress programmed cell death. The dashed oval from panels B and E is reproduced here to show that the eye size is similar to the hid^Ala5^ baseline. (G and H) Western analysis of Drk immunoprecipitation. (G) Drk was immunoprecipitated with anti-Drk 12201 antiserum and blotted with Ack (top panel) and Drk (bottom panel) antibodies. Ack co-precipitates with Drk from extracts prepared from S2 cells expressing endogenous Ack (S2 lane) as well as Ack overexpressing S2 cells (Ack OE lane). Ack knockdown by RNAi eliminates the immunoreactive band, confirming that it is Ack (Ack RNAi lane). (H) Drk was immunoprecipitated from Ack overexpressing S2 cells using two different Drk antisera, 12201 (01) and 12203 (03), and blotted with an anti-phosphotyrosine antibody (4G10). An immunoreactive band corresponding to phospho-Ack is indicated (P-Ack). No immunoreactivity is detected where Drk is predicted to run (P-Drk).

Drk is an adaptor protein made up of SH2 and SH3 domains and is the fly ortholog of vertebrate GRB2 and *C. elegans* Sem5. In both vertebrates and *C. elegans*, these proteins have been shown to interact with their Ack equivalents to negatively regulate EGF receptor signaling [50], [53]. We find in the absence of Ack overexpression, Drk RNAi does not influence hid or hid^Ala5^ induced apoptosis as assessed by modification of eye size (compare Figure 7A to 7D and 7B to 7E). Expression of Ack suppresses hid^Ala5^ induced cell death (compare Figure 7B to 7C), and remarkably this suppression can be eliminated by RNAi knockdown of Drk (compare Figure 7B and 7C to 7F). We conclude from these experiments that Ack requires Drk function for transmission of its anti-apoptotic signal. Drk immunoprecipitation followed by western analysis shows that Drk and Ack exist in a physical complex, and this interaction can be enhanced by increasing Ack expression levels (Figure 7G). We further find that the Ack that co-precipitates with Drk is tyrosine phosphorylated, as assessed by western analysis, but we are unable to detect any tyrosine phosphorylation on Drk itself (Figure 7H). Taken together, these data support a model in which Drk is not an Ack substrate, but serves as an adapter protein that functions to position Ack to receive and propagate anti-apoptotic signals.

### Yorkie and Ack Genetically Interact to Promote Proliferation and Block Apoptosis

Yorkie (yki) is the Ack interacting protein that has the second highest Mascot score. Yki has not been previously identified as an Ack family member binding protein, but is an attractive candidate due to its role as a transcriptional co-activator that interacts with the TEAD/TEF family protein Scalloped to control expression of proliferative (Cyc E) and anti-apoptotic genes (DIAP) [54], [55]. Nuclear yki is shuttled to the cytoplasm by the FERM-domain containing protein Expanded [56]. Cytoplasmic yki is subject to inactivation by the Salvador-Warts-Hippo signaling pathway, which functions to regulate organ size [57]. Yki phosphorylation by the serine/threonine kinase Warts leads to yki interaction with cytoplasmic 14-3-3 proteins and yki sequestration from the nucleus [58]. Since yki is a transcriptional co-activator of both proliferative and anti-apoptotic genes, it is tempting to speculate that Ack functions to promote the nuclear localization of yki to enhance transcriptional activation of yki target genes. We conducted a series of experiments to test this hypothesis and further characterize the interactions between Ack and yki.

**Figure 8:**
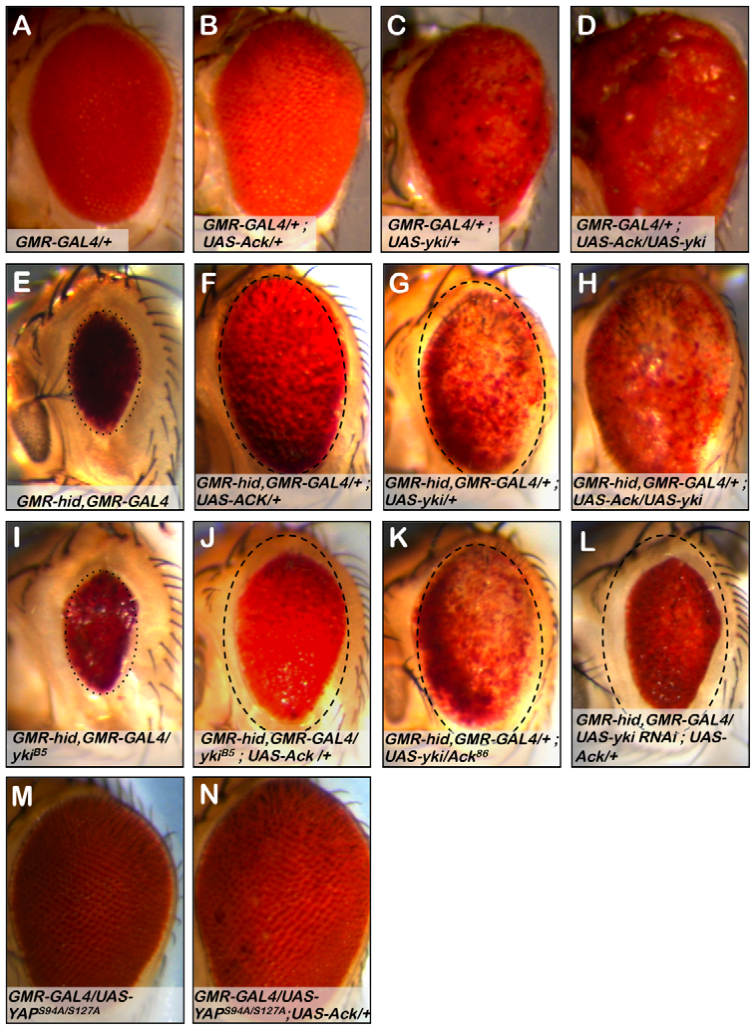
Ack and Yorkie genetically interact. The eye size assay was employed to assess the involvement of Yorkie (yki) in Ack suppression of apoptosis. Genotypes are indicated on each panel. (A) *GMR-Gal4* control eye. (B) Ack overexpression does not modify *GMR-Gal4* roughness, but does result in a slightly increased eye size. (C) Yki overexpression results in a disorganized eye that is overgrown. (D) Ack and yki overexpression yields a massively overgrown eye. (E) Hid small eye control phenotype. (F) Ack overexpression suppresses hid induced PCD. The eye is outlined with a dashed oval that has been reproduced in panels G and J–L to aid in size comparisons. (G) Yki overexpression in the hid background not only results in hid PCD suppression, but also shows a reduction in pigmentation. (H) Yki and Ack overexpression in a hid background results in further PCD suppression than either transgene alone, but still shows loss of pigmentation. (I) *Yki* single copy loss results in an eye that is a similar size as the hid control phenotype, shown by a dotted oval. (J) Single copy loss of *yki* reduces the ability of Ack to suppress hid induced PCD. (K) Single copy loss of *Ack* does not modify yki suppression of PCD. (L) Knockdown of yki by RNAi further interferes with the ability of Ack to suppress hid induced apoptosis compared to *yki* single copy loss. (M) Exogenous expression of human YAP^S94A/S127A^ does not affect eye size. (N) Combined expression of YAP^S94A/S127A^ and Ack results in an increased eye size.

We have shown that expression of Ack in the *Drosophila* eye blocks native apoptosis (Figure 3C) and produces a slightly larger and mildly rough eye (Figure 8B). Expression of yki in the eye produces a strong overgrowth phenotype in which eye tissue protrudes out of the normal curvature of the eye (Figure 8C). Co-expression of Ack and yki reveals a synergistic effect, producing massive overgrowth (Figure 8D). We draw two conclusions from these results. First, Ack and yki expression in the eye result in different phenotypes, suggesting that Ack may not activate yki transcriptional activity. Second, Ack activity is not limited to prevention of apoptosis, but can also enhance proliferation when combined with proliferative signals.

Next we sought to determine if Ack and yki expression resulted in similar suppression of hid induced programmed cell death in the eye. We find that Ack and yki result in similar magnitudes of suppression of hid small eye size (Figure 8E–8G); however, yki expression in the hid background produces eyes displaying a loss of eye pigment whereas Ack expression results in uniformly pigmented eyes. Co-expression of Ack and yki in the *GMR-hid* background further suppresses the hid small eye phenotype, but still results in an eye with comparable pigment loss to yki suppression of hid alone (Figure 8H). The fact that we observe different results for Ack (Figure 8F) versus yki (Figure 8G) suppression of the hid induced small eye phenotype suggests that Ack suppresses apoptosis via a mechanism that is independent of yki mediated transcriptional co-activation.

### Ack Does Not Promote Yki Nuclear Translocation or Transcriptional Activation of Yki Targets

Based on our data, it is unlikely that Ack enhances yki nuclear localization and subsequent activation of yki transcriptional targets. To confirm this we assessed the effect that Ack has on yki subcellular localization. Ack-mCherry expressed in R-cells is excluded from the nucleus and localizes throughout the cytoplasm. The highest concentrations of Ack are at apical membrane surfaces and in puncta near the nuclear envelope and within axons (Figure S4A). Yki-GFP expressed in R-cells has a localization pattern similar to Ack-mCherry, exhibiting nuclear exclusion with cytoplasmic, apical membrane and axonal puncta localization; however, the highest levels are seen ringing the nucleus with puncta apparent on or near the nuclear envelope (Figure S4B). Co-expression of Ack-mCherry and yki-GFP reveals overlapping expression patterns in the apical membrane regions and both axon and nuclear localizing puncta (Figure S4C–S4E). Co-expression of Ack and yki does not result in increased nuclear localization of either protein but does increase the yki localization to puncta surrounding the nucleus. Consistent with these data, we detected no increases in beta-galactosidase expression in the yki transcriptional activity reporter line ex^697^ when Ack is overexpressed (Figure S5A–S5F). These data are consistent with Ack and yki participating in physical interactions outside of the nucleus that do not lead to nuclear import of either protein or transcriptional up-regulation of yki targets.

### Yki Regulates Ack Anti-Apoptotic and Proliferative Functions

We tested the effect of protein dosage on the ability of Ack or yki to suppress hid induced apoptosis. Single copy loss of yki dominantly suppresses Ack anti-apoptotic function (compare Figure 8F to 8J), while single copy loss of Ack has no effect on the ability of yki to suppress the hid induced small eye phenotype (compare Figure 8G to 8K). Single copy loss of yki did not noticeably modify the hid induced small eye in the absence of Ack suggesting that yki loss is specifically affecting Ack function (Compare Figure 8E to 8I). Finally, we employed yki RNAi to further reduce the levels of yki in the eye. Yki RNAi in the *GMR-hid* background resulted in flies that die as pharate adults. Expression of exogenous Ack in this background rescues lethality and produces eye sizes smaller than yki single copy loss (Figure 8l). Taken together these data support a model in which Ack anti-apoptotic function is activated by yki protein.

Yes associated protein (YAP) is the vertebrate homolog of yki. Mutant versions of YAP have been generated and studied in the *Drosophila* eye. The YAP S127A mutation prevents phosphorylation by LATS family kinases, thereby increasing YAP nuclear localization. The YAP S94A mutation disrupts the binding of YAP to TEAD family DNA binding proteins. Expression of YAP^S127A^ in the fly eye leads to an overproliferation phenotype, while expression of the double mutant YAP^S94A/S127A^ produces eyes that are normal in appearance due to an inability to bind Sd and activate YAP/yki target gene transcription [59]. We obtained YAP^S94A/S127A^ and found that co-expression of YAP^S94A/S127A^ with Ack leads to an increase in eye size (compare Figure 8M and 8N), indicating that YAP can also genetically interact with Ack to enhance proliferation even when it cannot serve as a transcriptional co-activator with Scalloped.

## Discussion

*Drosophila melanogaster* has two Ack family members: Ack and PR2. We have found that Ack possesses anti-apoptotic properties, while PR2 either does not possess anti-apoptotic properties or requires activators not present in our assay system. While Ack may appear to be more closely related to vertebrate TNK1 because both proteins lack a CRIB domain, Ack is most similar to vertebrate ACK1 based on sequence identity of all shared protein domains. Additionally we find that many of the proteins that interact with ACK1 also interact with fly Ack. Therefore, we feel that the conclusions drawn in this study will likely be applicable to vertebrate ACK1 function.

We have shown that Ack function can suppress programmed cell death induced by hid or rpr expression in the developing eye and wing discs. Our overexpression studies reveal that Ack kinase activity is required for suppression of apoptosis induced by hid but is unnecessary for suppression of rpr-induced apoptosis. We further show that Ack loss of function enhances cell death induced by expression of both of these genes and determine that Ack is critically required for the survival of rpr expressing eye tissue. The molecular mechanisms underlying these differential requirements are not known. Hid and rpr are known to function in a multimeric protein complex on the mitochondria outer membrane to promote apoptosis [60]. Both hid and rpr are able to stimulate apoptosis by competing with initiator and effector caspases for DIAP binding, but rpr additionally induces DIAP auto-ubiquitination leading to DIAP degradation. While Ack kinase activity may be important for aspects of hid complex regulation, it is tempting to speculate that the UBA domains of Ack may play a critical role in the modulation of DIAP or rpr ubiquitination and stability.

EGF receptor signaling has been shown to activate ACK1 in vertebrates, and we find that EGF signaling enhances the anti-apoptotic function of Ack in *Drosophila*. ACK1 negatively regulates EGF receptor signaling by stimulating endocytosis of activated receptor complexes. We have evidence supporting that *Drosophila* Ack, in conjunction with SH3PX1, functions in a similar manner, which will be described elsewhere. In *Drosophila*, EGF signaling is anti-apoptotic through the activation of MAPK, which phosphorylates and inactivates hid. If Ack affected apoptosis exclusively through attenuation of EGF signaling, then we would expect that Ack loss of function would be anti-apoptotic while gain of function would be pro-apoptotic, which is opposite to what we observe. By using the hid^Ala5^ mutant, we demonstrate that Ack does not modulate programmed cell death through activation of MAPK.

The anti-apoptotic function of Ack is surprisingly robust compared to other proteins that we have tested. Our studies show that activity of the kinase domain contributes to Ack anti-apoptotic function. Based on this, we conclude that Ack propagates anti-apoptotic signals by phosphorylating downstream targets. Several ACK1 substrates have been identified that are attractive candidates for the regulation of programmed cell death: the putative tumor suppressor WWOX, the apoptosis inhibiting protein kinase AKT and the caspase-cleaved ubiquitin E3 ligase NEDD4. Akt1 loss and gain of function alleles (Figure S3) and Nedd4 RNAi (Figure 6K) fail to significantly modify hid induced small eye phenotypes. Wwox RNAi is able to suppress hid induced apoptosis but not nearly as robustly as Ack expression (Figure S6). Since ACK1 mediated phosphorylation of WWOX leads to WWOX destruction, we would predict that Wwox RNAi would phenocopy Ack expression in our assay system, but it is unable to reproduce the magnitude of Ack anti-apoptotic function. This does not rule out Wwox as an anti-apoptotic substrate target of Ack, but it demonstrates that Wwox is not the only cell death relevant substrate of Ack. It is worth noting that hid and rpr act fairly late within the programmed cell death pathway, being just a step upstream of initiator caspase activation. Therefore, Ack must target substrates that have activities influencing hid, rpr or events at the level of caspase activation.

We identified several Ack physically interacting proteins using a tandem affinity purification strategy. Many of these have vertebrate homologs that have been previously determined to interact with ACK1. We found that Drk and yki have the most pronounced effect on Ack's anti-apoptotic properties and chose to further characterize their contribution to Ack signaling. Drk is the fly ortholog of vertebrate GRB2, which has previously been described as an ACK1 and TNK1 interacting protein. In the case of TNK1, GRB2 is tyrosine phosphorylated by TNK1, which disrupts the ability of GRB2/SOS complexes to activate Ras [26]. This does not appear to be the case for *Drosophila* Ack, because even though Drk forms a complex with tyrosine phosphorylated Ack, we find no evidence of tyrosine phosphorylation on Drk. Rather, our data support that Drk association with Ack is required for Ack anti-apoptotic properties. We propose that Drk SH3 domains likely interact with PXXP motifs in the C-terminal half of Ack similar to the interaction described in vertebrates [50]. This interaction could then lead to the recruitment of Ack into protein complexes required for Ack activation.

Yki is a transcriptional co-activator that regulates expression of genes with proliferative and anti-apoptotic functions. The vertebrate homolog of yki is Yes Associated Protein (YAP), which has not previously been identified as an ACK1 or TNK1 interacting protein. Yki and YAP studies have focused primarily on the pathways that regulate their function as transcription factors. Given the role of yki and YAP in transcriptional control of proliferative and anti-apoptotic genes, it would seem likely that Ack activity leads to enhancement of yki function. However, this does not appear to be the case because Ack overexpression does not lead to increased yki nuclear localization or increased expression of yki target genes. Rather, our data indicate that yki directly interacts with Ack in the cytoplasm and functions to regulate Ack activity. In support of this, we have found that Ack colocalizes with yki, and *yki* dosage reduction suppresses Ack anti-apoptotic function. Yki contains two WW domains, which may interact with conserved PPXY motifs that are present in the region flanked by the SH3 and UBA domains of Ack family members. In vertebrates, these PPXY motifs have been shown to interact with WWOX, which also contains two WW domains [16]. Further studies are required to define how yki and Ack interact.

Yki expression in the fly eye produces an overgrowth phenotype that is indicative of its role in regulating proliferation. Ack overexpression produces a slightly larger eye due to inhibition of apoptotic events that occur during normal eye development. Simultaneous expression of yki and Ack results in a synergistic effect that produces enormous eyes. These results reveal that in addition to anti-apoptotic function, Ack can also enhance proliferation. This illustrates how increased Ack signaling could contribute to cancer when coupled to proliferative signals. Indeed, our results are consistent with recent reports of ACK1 activating somatic mutations [12] and gene amplification [13] being associated with human cancers. At present the key anti-apoptotic substrates of Ack and their mechanisms of action remain to be determined. With their discovery will come a better understanding of Ack signaling and potentially new targets for cancer interventions.

## Materials and Methods

### Fly Lines

Standard *Drosophila* genetic technique was used for all crosses and transgenesis. All flies were raised at 25°C with 70% relative humidity. Fly lines used are as follows: Oregon R, *GMR-Gal4/CyO*, *GMR-hidG1/CyO*, *GMR-p35*, *P{lacW}Gap1/TM3*, *argos^Δ7^/TM3*, *UAS-Dcr-2/CyO*, *UAS-Dcr-2*, *P{PZ}Akt1^04226^/TM3*, *GMR-rpr/TM6B*, *UAS-yki-GFP* (Bloomington Stock Center); *UAS-PR2* and *Ack^86^/TM3* (a gift from Nicholas Harden, Simon Fraser University); *GMR-hid^Ala5^/CyO* (a gift from Andreas Bergmann, M.D. Anderson Cancer Center); *UAS-yki* and *FRT42*, *yki^B5^/CyO* (a gift from Nic Tapon, London Research Institute); *UAS-YAP^S127A^* and *UAS-YAP^S94A/S127A^* (a gift from Zhi-Chun Lai, Pennsylvania State Univeristy); *rn-GAL4*, *UAS-rpr*, *tub-GAL80^ts^/TM6B*, *GAL80* (a gift from Ken Irvine, Rutgers University); *UAS-PR2 RNAi*, *UAS-Ack RNAi*, *UAS-SH3PX1 RNAi*, *UAS-CG4169 RNAi*, *UAS-Gale RNAi*, *UAS-Drk RNAi*, *UAS-HSP 26 RNAi*, *UAS-HSP 23 RNAi*, *UAS-HSP 83 RNAi*, *UAS-yki RNAi*, *UAS-14-3-3 epsilon RNAi*, *UAS-Magi RNAi*, *UAS-Dock RNAi*, *UAS-Nedd4 RNAi*, *UAS-Wwox RNAi* (Vienna *Drosophila* RNAi Center, VDRC).

UAS-Ack constructs were produced by sub-cloning Ack from pMT/V5-Ack plasmid (a gift from Jack Dixon, UCSD) into EcoRI/XhoI sites in the pUASt vector. Transgenic flies (*UAS-Ack/TM6B*, *UAS-Ack^K156A^/TM6B* and UAS-Ack-mCherry) were made by microinjecting pUASt-Ack DNA constructs into *yw* flies. *GMR-hid* and *GMR-hid^Ala5^* were recombined with *GMR-Gal4* to aid in the *Drosophila* eye size assays.

### Testis Analysis

Newly eclosed males were dissected in PBS and transferred to PBX (PBS+0.1% Triton X-100) containing 4% formaldehyde for 20 minutes. Testis were washed with PBX, incubated with anti-cleaved caspase-3 (cell signaling, 1∶75) overnight at 4°C followed by anti-rabbit Alexa 488 (1∶500, Invitrogen) secondary antibody for 3 hours. F-actin was stained using 1 unit of Alexa 568 phalloidin (Invitrogen) and nuclei were stained with Hoechts used at 1∶10,000 dilution (Thermo Scientific). Samples were mounted in VECTASHIELD and images were acquired using a Disk Scanning Unit-enabled Olympus BX61 microscope equipped with a Hamamatsu DCMA-API camera.

### Hid and Hid^Ala5^ Eye Cell Death Modifier Assay

*GMR-Gal4*, *GMR-hid*/CyO or *GMR-Gal4*, *GMR-hid^Ala5^/CyO* virgin females were crossed with male flies of various genotypes. Color images were taken by removing the heads and mounting them with rubber cement onto 1 µl micropipettes and imaged submerged in water using a Zeiss Discovery.V12 dissecting microscope equipped with a color camera and a plan S 1.0X FWD 81 mm objective. All images were acquired and processed identically. Images were taken at 85× magnification, cropped & rotated using Adobe Photoshop and then scaled uniformly using Microsoft PowerPoint.

### Eye Disc Analysis

Images were acquired using a Zeiss LSM 710 confocal microscope. For immuno and direct fluorescence, 3^rd^ instar larva were dissected in PBS and fixed in 4% paraformaldehyde for 30 minutes. Eye discs expressing GFP and mCherry fusion proteins were washed prior to mounting in VECTASHIELD (Vector Laboratories) and fluorescent imaging. For analysis of non-tagged proteins, the eye discs were blocked in PBTN (10% normal goat serum in PBT (PBS+0.5% Triton X-100)) and incubated overnight in PBTN containing antibodies directed against Ack (JCD2, 1∶1000) and beta-galactosidase (40-1a, 1∶50, Developmental Studies Hybridoma Bank, University of Iowa) followed by one hour incubations with goat anti-mouse Alexa 568 (1∶500) and goat anti-rabbit Alexa 488 (1∶500) secondary antibodies.

For TUNEL and BrdU analysis, 3^rd^ instar larva imaginal discs were dissected in PBS and incubated in BrdU incorporation solution (5 µl/ml BrdU (Sigma) in PBS) for 1 hour at room temperature (RT). Eye discs were then washed with PBS and fixed with 4% paraformaldehyde for 30 minutes. The samples were blocked overnight at 4°C with PBTN.

TUNEL labeling: Eye discs were incubated in 99 mM Na-Citrate+0.1% Trition X-100 for 30 minutes at 65°C, washed and incubated in TUNEL assay solution (In Situ Cell Death Detection Kit, Roche) for 1 hour at 37°C rotating in the dark. Eye discs were then washed in PBT and once in PBS then treated with 2N HCl for 30 minutes and neutralized with 100 mM Borax for 2 minutes. They were then washed with PBT and re-blocked with PBTN for 1 hour at RT.

BrdU Staining: tissue was incubated overnight at 4°C in anti-BrdU primary antibody (1∶20, G3G4, Developmental Studies Hybridoma Bank, University of Iowa), washed and incubated for 3 hours at RT with goat anti-mouse Alexa 568 (1∶500) secondary antibody. Eye discs were washed twice with PBT, twice with PBS and then mounted in VECTASHIELD.

### Wing Disc Analysis

*rn-GAL4*, *UAS-rpr*, *tub-GAL80^ts^/TM6B*, *GAL80* flies were crossed to *yw*, *UAS-Ack*, *UAS-Ack^K156A^* or *Ack^86^* and the progeny were raised at 18°C until third instar larva appeared. Male third instar larva were isolated and transferred to new vials that contained approximately 1 ml of mechanically churned food. Vials were then placed in a 30°C water bath for 3 hours and then returned to 18°C for four hours. Wing discs were dissected, fixed and subjected to TUNEL analysis as described above.

### Pupa Retina Analysis

Crosses were established at 22°C and larvae allowed to pupate to 42% pupal development. Retinas were dissected in standard saline buffer (2 mM KCl, 128 mM NaCl, 4 mM MgCl_2_, 1.8 mM CaCl_2_, 36 mM sucrose, 5 mM HEPES pH 7.1). Immediately after dissection, retinas were immersed in fixative (10 mM periodate, 75 mM lysine, 2% paraformaldehyde in 1× PBS) with 0.05% saponin for 20 minutes. Following fixation, retinas were washed in PBX and incubated with mouse anti-Armadillo (1∶30; DSHB) and Alexa 568 phalloidin (Molecular Probes) with 5% goat serum overnight at 4°C. Retinas were then washed in PBST and incubated in Alexa 646 (Molecular Probes) and Alexa 488 conjugated secondary antibodies for 3 hours at room temperature. Retinas were then washed with PBST and mounted in mounting medium (0.25% n-propyl gallate, 50% glycerol in PBS). Confocal images were taken and the images were inverted and printed. For easier analysis the pigment cells were false colored.

### Purification of Ack Interacting Proteins and Mass Spectrometry

Schneider 2 (S2) *Drosophila* cells were transfected with pMT/V5-6xHis-Flag-Ack and pCoHygro in Schneider's S2 medium (Invitrogen). Stably transfected cells were obtained by hygromycin selection (200 µg/ml, Invitrogen). Both control S2 cells (non-transfected) and 6xHis-Flag-Ack expressing S2 cells were subjected to the following purification procedure and mass spectrometry analysis. Cells were grown in a liter spinner flask and induced with 0.7 mM CuSO_4_ for 1 day. Cells were harvested and suspended in RIPA buffer (150 mM NaCl, 1% Igepal CA-630, 0.5% sodium deoxycholate, 0.1% SDS, 50 mM Tris pH 8.0, .2 mM sodium orthovanadate, 10 mM NaF, 0.4 mM EDTA pH 8, 10% glycerol) then lysed by sonication. 6xHis-Flag Ack was captured using Flag M2 resin (Sigma) and Ack was eluted with 100 µg/ml 3× flag peptide (Sigma). A second level of purification was achieved by adding a Phospho-tyrosine resin (protein A with cross-linked 4G10 antibody (Millipore)) to the flag elution. The final elution was with 100 mM triethanolamine (TEA) pH 11.5 and dried using a speedvac.

To prepare samples for mass spectrometry, purified proteins were resuspended in 50 mM ammonium bicarbonate with 0.2% RapiGest (Waters). 5 mM tris (2-carboxyethyl) phosphine was added and the sample was heated at 56°C for 30 minutes. After heating, 15 mM Iodoacetamide was added and vortexed for 1 hour in the dark. 1 µg trypsin was added to digest the protein overnight at 37°C. 0.5 ul of concentrated HCl was added to degrade the RapiGest. The sample was then centrifuged and dried using a speedvac and subsequently resuspended in 5% acetonitrile, 0.1% trifluoroacetic acid prior to liquid chromatography and electrospray ionization-MS/MS analysis. An Agilent 1100 nano-HPLC system was used to separate peptides using Zorbax C_18_ trap and 75 um×150 mm capillary columns. Peptides were eluted with a gradient of increasing acetonitrile in 0.1% formic acid at a flow of 300 nL/min and injected into a Thermo LTQ-Orbitrap using a nanoelectrospray source. The Orbitrap was operated in data-dependent tandem MS mode with one MS scan followed by 3 tandem MS scans and a dynamic exclusion window of two minutes. Data were searched against a database of *Drosophila melanogaster* protein sequences using both Sorcerer (Sage-N Research) and Mascot (MatrixScience) search software. Similar results were obtained with both. Database searches were set with a mass tolerance of 25 ppm, full tryptic cleavage, one allowed mis-cleavage, and carbamidomethyl cysteine modification. Identified proteins were considered specific Ack binding partners only if the following stringent criteria were met: no peptides from the protein were observed in the control sample; at least 3 unique peptides were identified with Mascot ion scores above the designated threshold significance value; and Mascot protein scores were above 100.

### Immunoprecipitation and Western Analysis

S2 cells and S2 cells expressing exogenous Ack were grown to confluency, counted and plated in a 6-well dish containing S2 medium at a concentration of 1×10^6^ cells/1 ml/well. Ack dsRNA (15 µg) was added to a well with S2 cells and incubated for 45 minutes before the addition of 2 ml S2 media supplemented with 10% FBS to all wells. After 24 hours, 0.7 mM CuSO_4_ was added to the Ack overexpressing line to induce Ack expression. The cells were harvested after an additional 48 hours and lysed in RIPA buffer by sonication. Cleared lysate was achieved from a tandem centrifugation procedure of 10 minutes at 4°C on a tabletop centrifuge and then transferred to an ultracentrifuge for 30 minutes at 100,000×g at 4°C. The lysates were incubated with anti-Drk antibody (a gift from Efthimios Skoulakis, Alexander Fleming Biomedical Sciences Research Center, Greece) on ice for 1 hour before adding 50 µl slurry of immobilized protein A resin (G-Biosciences) for 1½ hours at 4°C with continuous rotation. The samples were washed with RIPA buffer.

Drk IP samples were eluted in SDS-PAGE sample buffer and subjected to SDS-PAGE. The protein was transferred to an immobilon-P transfer membrane (Millipore) and blocked for 1 hour with 5% milk. Drk and Ack were detected using anti-Drk 12201 antibody (1∶3,000, overnight) and anti-JCD2 (1∶1,500, 1 hour; [61]) primary antibodies and goat anti-rabbit secondary antibody (1∶10,000) for 1 hour and detected with ECL detection reagent (Thermo Scientific).

## Supporting Information

Figure S1 PR2 does not modify the small eye phenotypes induced by hid or rpr. The eye size assay was used to assess the ability of PR2 overexpression and knockdown to modify both hid and rpr induced programmed cell death. (A) Hid expression in a GAL4 background produces a small eye phenotype. Overexpression of PR2 (B) or RNAi mediated knockdown of PR2 (C) in the *GMR-hid*, *GMR-GAL4* background does not result in modification of eye size. The dotted oval in panel A has been reproduced in panels B and C to aid in comparison. (D) Rpr expression in a GAL4 background also produces a small eye phenotype. Similarly, overexpression of PR2 (E) or RNAi mediated knockdown of PR2 (F) in the *GMR-GAL4*, *GMR-rpr* background does not substantially modify eye size. The dashed oval in panel D has been reproduced in panels E and F to aid in comparison.
(TIF)

Figure S2 Ack manipulation modifies rpr induced programmed cell death in the wing disc. The TUNEL assay was used to assess the effect of Ack transgenes or alleles on rpr induced programmed cell death. (A–D) TUNEL positive cells are labeled in green and the genotypes are indicated in each panel. (A′–D′) the TUNEL positive cell images from A–D are superimposed on bright field micrographs of the eye disc. (A) *UAS-rpr* induces programmed cell death in the wing disc. (B) Ack overexpression results in fewer TUNEL positive cells. (C–D) Expression of kinase inactive Ack or Ack gene dosage reduction shows an increase in TUNEL positive cells.
(TIF)

Figure S3 Akt1 does not modify the hid small eye phenotype. The eye size assay was used to assess the ability of Akt1 loss and gain of function to modify hid induced programmed cell death. Genotypes are indicated in each panel. To aid in eye size comparisons, the dotted oval in panel A is reproduced in panels B–D, the dashed oval in panel E is reproduced in panels F–H and the dashed oval in panel I is reproduced in panels J–L. (A–D) The *GMR-hid*, *GMR-GAL4* genetic background combined with (B) Akt1 overexpression, (C) introduction of a loss of function allele Akt1^04226^ or (D) overexpression of the constitutively activated mutant Akt1^T342D/S505D^. (E–H) The *GMR-hid^Ala5^*, *GMR-GAL4* genetic background combined with (F) Akt1 overexpression, (G) the loss of the function allele Akt1^04226^ or (H) constitutively activated Akt1^T342D/S505D^ overexpression. (I–L) Ack overexpression in the *GMR-hid^Ala5^*, *GMR-GAL4* genetic background combined with (J) Akt1 overexpression, (K) the loss of the function allele Akt1^04226^ or (L) constitutively activated Akt1^T342D/S505D^ overexpression.
(TIF)

Figure S4 Ack-mCherry and yki-GFP subcellular localization in R-cells. Single plane confocal images show the subcellular localization of Ack-mCherry and yki-GFP expressed individually (A and B) or simultaneously (C–E) in third instar R-cells. Nuclei appear as open ringed structures in all panels. (A) Ack-mCherry is nuclear excluded and is found in the cytoplasm and in numerous cytoplasmic puncta. (B) Yki-GFP is also largely nuclear excluded and more diffusely distributed throughout the cytoplasm compared to Ack. (C–E) Simultaneous expression of Ack-mCherry (C) and yki-GFP (D) does not lead to increased nuclear localization of either protein, but does induce yki to co-localize into puncta with Ack. (E) An overlay of panels C and D showing Ack-mCherry (red) and yki-GFP (green) localization patterns.
(TIF)

Figure S5 Ack expression does not induce transcription of yki targets. Confocal images of third instar eye discs analyzed for expression of Ack and beta-galactosidase driven by the ex-lacZ enhancer trap line ex^697^ in the absence (A–C) or presence (D–F) of Ack overexpression (posterior is down). The dashed line indicates the position of the morphogenetic furrow (MF). (A) Beta-galactosidase expression pattern in the absence of Ack overexpression shows similar levels of labeling posterior and anterior of the MF. (B) Ack expression is higher posterior of the MF. (C) An overlay of panels A and B showing beta-galactosidase (green) and Ack (red) expression. (D) Beta-galactosidase expression pattern in the presence of Ack overexpression again shows similar levels of labeling posterior and anterior of the MF. (E) Overexpression of Ack can be seen posterior to the MF due to GMR driven expression. (F) An overlay of panels D and E showing beta-galactosidase (green) and Ack (red) expression.
(TIF)

Figure S6 Suppression of hid induced small eye phenotypes by RNAi mediated knockdown of Wwox. The eye size assay was used to assess the ability of Wwox knockdown modify both hid and hid^Ala5^ induced programmed cell death. (A) Hid expression in a *GMR-GAL4* and *UAS-Dcr2* expressing background produces a small eye phenotype. (B) RNAi mediated knockdown of Wwox suppresses the small eye phenotype. A dotted oval is used to aid in comparison. (C) Ack expression produces a larger increase in eye size in a similar genetic background. (D) Hid^Ala5^ expression in a *GMR-GAL4* and *UAS-Dcr2* expressing background also produces a small eye phenotype. (E) RNAi mediated knockdown of Wwox fails to modify the eye size in the *GMR-hid^Ala5^*, *GMR-GAL4*; *UAS-Dcr2* background. A dashed oval is drawn for comparison. (F) Ack expression suppresses the small phenotype induced in a *GMR-hid^Ala5^*, *GMR-GAL4* background.
(TIF)

## References

[1] Manser E, Leung T, Salihuddin H, Tan L, Lim L (1993). A non-receptor tyrosine kinase that inhibits the GTPase activity of p21cdc42.. Nature.

[2] Yokoyama N, Miller WT (2003). Biochemical properties of the Cdc42-associated tyrosine kinase ACK1. Substrate specificity, authphosphorylation, and interaction with Hck.. J Biol Chem.

[3] Cotteret S, Chernoff J (2002). The evolutionary history of effectors downstream of Cdc42 and Rac.. Genome Biol.

[4] Sem KP, Zahedi B, Tan I, Deak M, Lim L (2002). ACK family tyrosine kinase activity is a component of Dcdc42 signaling during dorsal closure in Drosophila melanogaster.. Mol Cell Biol.

[5] Shen F, Lin Q, Gu Y, Childress C, Yang W (2007). Activated Cdc42-associated kinase 1 is a component of EGF receptor signaling complex and regulates EGF receptor degradation.. Mol Biol Cell.

[6] Chan W, Tian R, Lee YF, Sit ST, Lim L (2009). Down-regulation of Active ACK1 Is Mediated by Association with the E3 Ubiquitin Ligase Nedd4-2.. J Biol Chem.

[7] Lin Q, Wang J, Childress C, Sudol M, Carey DJ (2010). HECT E3 ubiquitin ligase Nedd4-1 ubiquitinates ACK and regulates epidermal growth factor (EGF)-induced degradation of EGF receptor and ACK.. Mol Cell Biol.

[8] Yang W, Lin Q, Zhao J, Guan JL, Cerione RA (2001). The nonreceptor tyrosine kinase ACK2, a specific target for Cdc42 and a negative regulator of cell growth and focal adhesion complexes.. J Biol Chem.

[9] Yang W, Lo CG, Dispenza T, Cerione RA (2001). The Cdc42 target ACK2 directly interacts with clathrin and influences clathrin assembly.. J Biol Chem.

[10] Mahajan K, Coppola D, Challa S, Fang B, Chen YA (2010). Ack1 mediated AKT/PKB tyrosine 176 phosphorylation regulates its activation.. PLoS ONE.

[11] Mahajan NP, Liu Y, Majumder S, Warren MR, Parker CE (2007). Activated Cdc42-associated kinase Ack1 promotes prostate cancer progression via androgen receptor tyrosine phosphorylation.. Proc Natl Acad Sci U S A.

[12] Prieto-Echague V, Gucwa A, Brown DA, Miller WT (2010). Regulation of Ack1 localization and activity by the amino-terminal SAM domain.. BMC Biochem.

[13] van der Horst EH, Degenhardt YY, Strelow A, Slavin A, Chinn L (2005). Metastatic properties and genomic amplification of the tyrosine kinase gene ACK1.. Proc Natl Acad Sci U S A.

[14] Howlin J, Rosenkvist J, Andersson T (2008). TNK2 preserves epidermal growth factor receptor expression on the cell surface and enhances migration and invasion of human breast cancer cells.. Breast Cancer Res.

[15] Liu Z, Adams HC, Whitehead IP (2009). The rho-specific guanine nucleotide exchange factor Dbs regulates breast cancer cell migration.. J Biol Chem.

[16] Mahajan NP, Whang YE, Mohler JL, Earp HS (2005). Activated tyrosine kinase Ack1 promotes prostate tumorigenesis: role of Ack1 in polyubiquitination of tumor suppressor Wwox.. Cancer Res.

[17] Bednarek AK, Laflin KJ, Daniel RL, Liao Q, Hawkins KA (2000). WWOX, a novel WW domain-containing protein mapping to human chromosome 16q23.3–24.1, a region frequently affected in breast cancer.. Cancer Res.

[18] Finnis M, Dayan S, Hobson L, Chenevix-Trench G, Friend K (2005). Common chromosomal fragile site FRA16D mutation in cancer cells.. Hum Mol Genet.

[19] Ried K, Finnis M, Hobson L, Mangelsdorf M, Dayan S (2000). Common chromosomal fragile site FRA16D sequence: identification of the FOR gene spanning FRA16D and homozygous deletions and translocation breakpoints in cancer cells.. Hum Mol Genet.

[20] Bednarek AK, Keck-Waggoner CL, Daniel RL, Laflin KJ, Bergsagel PL (2001). WWOX, the FRA16D gene, behaves as a suppressor of tumor growth.. Cancer Res.

[21] Bellacosa A, Kumar CC, Di Cristofano A, Testa JR (2005). Activation of AKT kinases in cancer: implications for therapeutic targeting.. Adv Cancer Res.

[22] Grossmann ME, Huang H, Tindall DJ (2001). Androgen receptor signaling in androgen-refractory prostate cancer.. J Natl Cancer Inst.

[23] Chen CD, Welsbie DS, Tran C, Baek SH, Chen R (2004). Molecular determinants of resistance to antiandrogen therapy.. Nat Med.

[24] Hoare K, Hoare S, Smith OM, Kalmaz G, Small D (2003). Kos1, a nonreceptor tyrosine kinase that suppresses Ras signaling.. Oncogene.

[25] Hoare S, Hoare K, Reinhard MK, Lee YJ, Oh SP (2008). Tnk1/Kos1 knockout mice develop spontaneous tumors.. Cancer Res.

[26] May WS, Hoare K, Hoare S, Reinhard MK, Lee YJ (2010). Tnk1/Kos1: a novel tumor suppressor.. Trans Am Clin Climatol Assoc.

[27] Lierman E, Van Miegroet H, Beullens E, Cools J (2009). Identification of protein tyrosine kinases with oncogenic potential using a retroviral insertion mutagenesis screen.. Haematologica.

[28] Gu TL, Cherry J, Tucker M, Wu J, Reeves C (2010). Identification of activated Tnk1 kinase in Hodgkin's lymphoma.. Leukemia.

[29] Zahedi B, Shen W, Xu X, Chen X, Mahey M (2008). Leading edge-secreted Dpp cooperates with ACK-dependent signaling from the amnioserosa to regulate myosin levels during dorsal closure.. Dev Dyn.

[30] Bellen HJ, Levis RW, Liao G, He Y, Carlson JW (2004). The BDGP gene disruption project: single transposon insertions associated with 40% of Drosophila genes.. Genetics.

[31] Arama E, Agapite J, Steller H (2003). Caspase activity and a specific cytochrome C are required for sperm differentiation in Drosophila.. Dev Cell.

[32] Santel A, Blumer N, Kampfer M, Renkawitz-Pohl R (1998). Flagellar mitochondrial association of the male-specific Don Juan protein in Drosophila spermatozoa.. J Cell Sci.

[33] Santel A, Winhauer T, Blumer N, Renkawitz-Pohl R (1997). The Drosophila don juan (dj) gene encodes a novel sperm specific protein component characterized by an unusual domain of a repetitive amino acid motif.. Mech Dev.

[34] Grether ME, Abrams JM, Agapite J, White K, Steller H (1995). The head involution defective gene of Drosophila melanogaster functions in programmed cell death.. Genes Dev.

[35] Bergmann A, Agapite J, McCall K, Steller H (1998). The Drosophila gene hid is a direct molecular target of Ras-dependent survival signaling.. Cell.

[36] White K, Grether ME, Abrams JM, Young L, Farrell K (1994). Genetic control of programmed cell death in Drosophila.. Science.

[37] Chen P, Nordstrom W, Gish B, Abrams JM (1996). grim, a novel cell death gene in Drosophila.. Genes Dev.

[38] Goyal L, McCall K, Agapite J, Hartwieg E, Steller H (2000). Induction of apoptosis by Drosophila reaper, hid and grim through inhibition of IAP function.. Embo J.

[39] Bergmann A, Yang AY, Srivastava M (2003). Regulators of IAP function: coming to grips with the grim reaper.. Curr Opin Cell Biol.

[40] Zachariou A, Tenev T, Goyal L, Agapite J, Steller H (2003). IAP-antagonists exhibit non-redundant modes of action through differential DIAP1 binding.. Embo J.

[41] Ready DF, Hanson TE, Benzer S (1976). Development of the Drosophila retina, a neurocrystalline lattice.. Dev Biol.

[42] Wolff T, Ready DF (1991). Cell death in normal and rough eye mutants of Drosophila.. Development.

[43] Srivastava M, Scherr H, Lackey M, Xu D, Chen Z (2007). ARK, the Apaf-1 related killer in Drosophila, requires diverse domains for its apoptotic activity.. Cell Death Differ.

[44] Fan Y, Bergmann A (2010). The cleaved-Caspase-3 antibody is a marker of Caspase-9-like DRONC activity in Drosophila.. Cell Death Differ.

[45] Smith-Bolton RK, Worley MI, Kanda H, Hariharan IK (2009). Regenerative growth in Drosophila imaginal discs is regulated by Wingless and Myc.. Dev Cell.

[46] Kurada P, White K (1999). Epidermal growth factor receptor: its role in Drosophila eye differentiation and cell survival.. Apoptosis.

[47] Grovdal LM, Johannessen LE, Rodland MS, Madshus IH, Stang E (2008). Dysregulation of Ack1 inhibits down-regulation of the EGF receptor.. Exp Cell Res.

[48] Sawamoto K, Taguchi A, Hirota Y, Yamada C, Jin M (1998). Argos induces programmed cell death in the developing Drosophila eye by inhibition of the Ras pathway.. Cell Death Differ.

[49] Gaul U, Mardon G, Rubin GM (1992). A putative Ras GTPase activating protein acts as a negative regulator of signaling by the Sevenless receptor tyrosine kinase.. Cell.

[50] Galisteo ML, Yang Y, Urena J, Schlessinger J (2006). Activation of the nonreceptor protein tyrosine kinase Ack by multiple extracellular stimuli.. Proc Natl Acad Sci U S A.

[51] Satoh T, Kato J, Nishida K, Kaziro Y (1996). Tyrosine phosphorylation of ACK in response to temperature shift-down, hyperosmotic shock, and epidermal growth factor stimulation.. FEBS Lett.

[52] Mahajan K, Mahajan NP (2010). Shepherding AKT and androgen receptor by Ack1 tyrosine kinase.. J Cell Physiol.

[53] Hopper NA, Lee J, Sternberg PW (2000). ARK-1 inhibits EGFR signaling in C. elegans.. Mol Cell.

[54] Wu S, Liu Y, Zheng Y, Dong J, Pan D (2008). The TEAD/TEF family protein Scalloped mediates transcriptional output of the Hippo growth-regulatory pathway.. Dev Cell.

[55] Zhang L, Ren F, Zhang Q, Chen Y, Wang B (2008). The TEAD/TEF family of transcription factor Scalloped mediates Hippo signaling in organ size control.. Dev Cell.

[56] Badouel C, Gardano L, Amin N, Garg A, Rosenfeld R (2009). The FERM-domain protein Expanded regulates Hippo pathway activity via direct interactions with the transcriptional activator Yorkie.. Dev Cell.

[57] Huang J, Wu S, Barrera J, Matthews K, Pan D (2005). The Hippo signaling pathway coordinately regulates cell proliferation and apoptosis by inactivating Yorkie, the Drosophila Homolog of YAP.. Cell.

[58] Oh H, Irvine KD (2008). In vivo regulation of Yorkie phosphorylation and localization.. Development.

[59] Zhao B, Ye X, Yu J, Li L, Li W (2008). TEAD mediates YAP-dependent gene induction and growth control.. Genes Dev.

[60] Sandu C, Ryoo HD, Steller H (2010). Drosophila IAP antagonists form multimeric complexes to promote cell death.. J Cell Biol.

[61] Clemens JC, Worby CA, Simonson-Leff N, Muda M, Maehama T (2000). Use of double-stranded RNA interference in Drosophila cell lines to dissect signal transduction pathways.. Proc Natl Acad Sci U S A.

