# Supplementary figures and images for: Drosophila Activated Cdc42 Kinase Has an Anti-Apoptotic Function
Source: PLoS Genet. 2012 May 17;8(5):e1002725. doi: 10.1371/journal.pgen.1002725 (PMC3355085; doi:10.1371/journal.pgen.1002725)

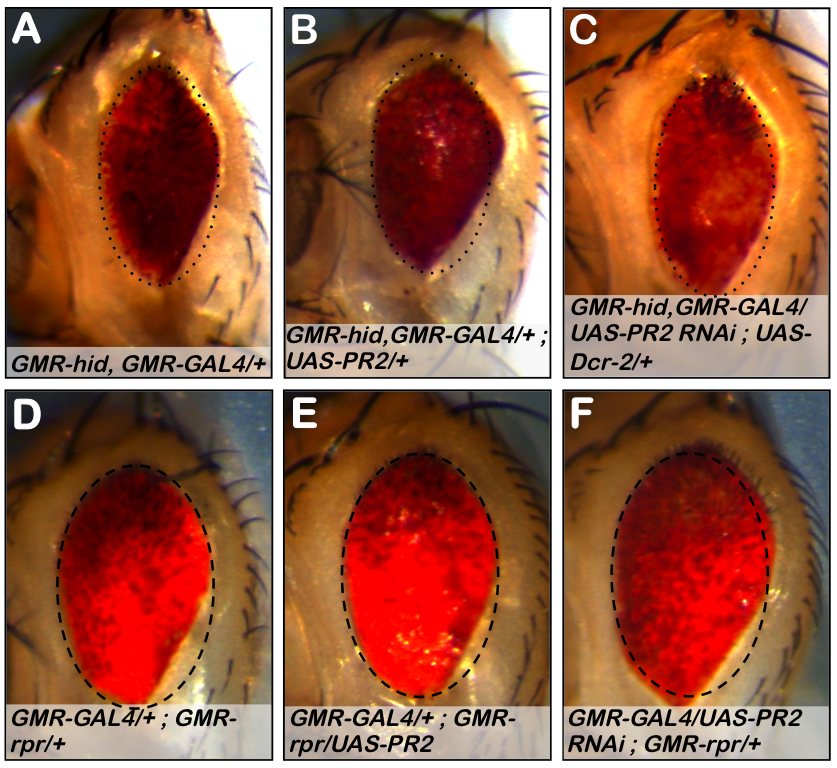

Supplement: Figure S1 — PR2 does not modify the small eye phenotypes induced by hid or rpr. The eye size assay was used to assess the ability of PR2 overexpression and knockdown to modify both hid and rpr induced programmed cell death. (A) Hid expression in a GAL4 background produces a small eye phenotype. Overexpression of PR2 (B) or RNAi mediated knockdown of PR2 (C) in the GMR-hid, GMR-GAL4 background does not result in modification of eye size. The dotted oval in panel A has been reproduced in panels B and C to aid in comparison. (D) Rpr expression in a GAL4 background also produces a small eye phenotype. Similarly, overexpression of PR2 (E) or RNAi mediated knockdown of PR2 (F) in the GMR-GAL4, GMR-rpr background does not substantially modify eye size. The dashed oval in panel D has been reproduced in panels E and F to aid in comparison. (TIF) [file pgen.1002725.s001.tif]

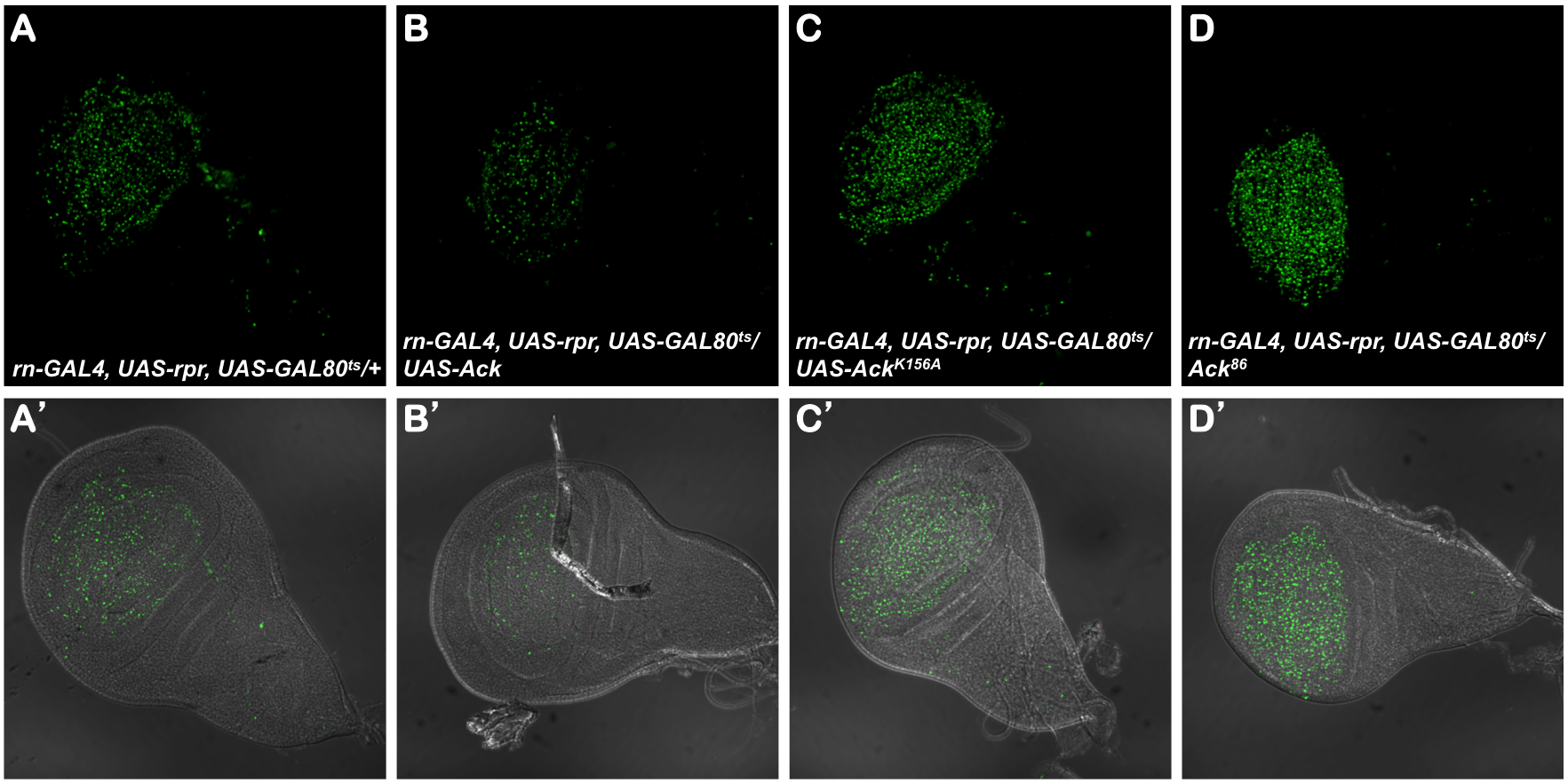

Supplement: Figure S2 — Ack manipulation modifies rpr induced programmed cell death in the wing disc. The TUNEL assay was used to assess the effect of Ack transgenes or alleles on rpr induced programmed cell death. (A–D) TUNEL positive cells are labeled in green and the genotypes are indicated in each panel. (A′–D′) the TUNEL positive cell images from A–D are superimposed on bright field micrographs of the eye disc. (A) UAS-rpr induces programmed cell death in the wing disc. (B) Ack overexpression results in fewer TUNEL positive cells. (C–D) Expression of kinase inactive Ack or Ack gene dosage reduction shows an increase in TUNEL positive cells. (TIF) [file pgen.1002725.s002.tif]

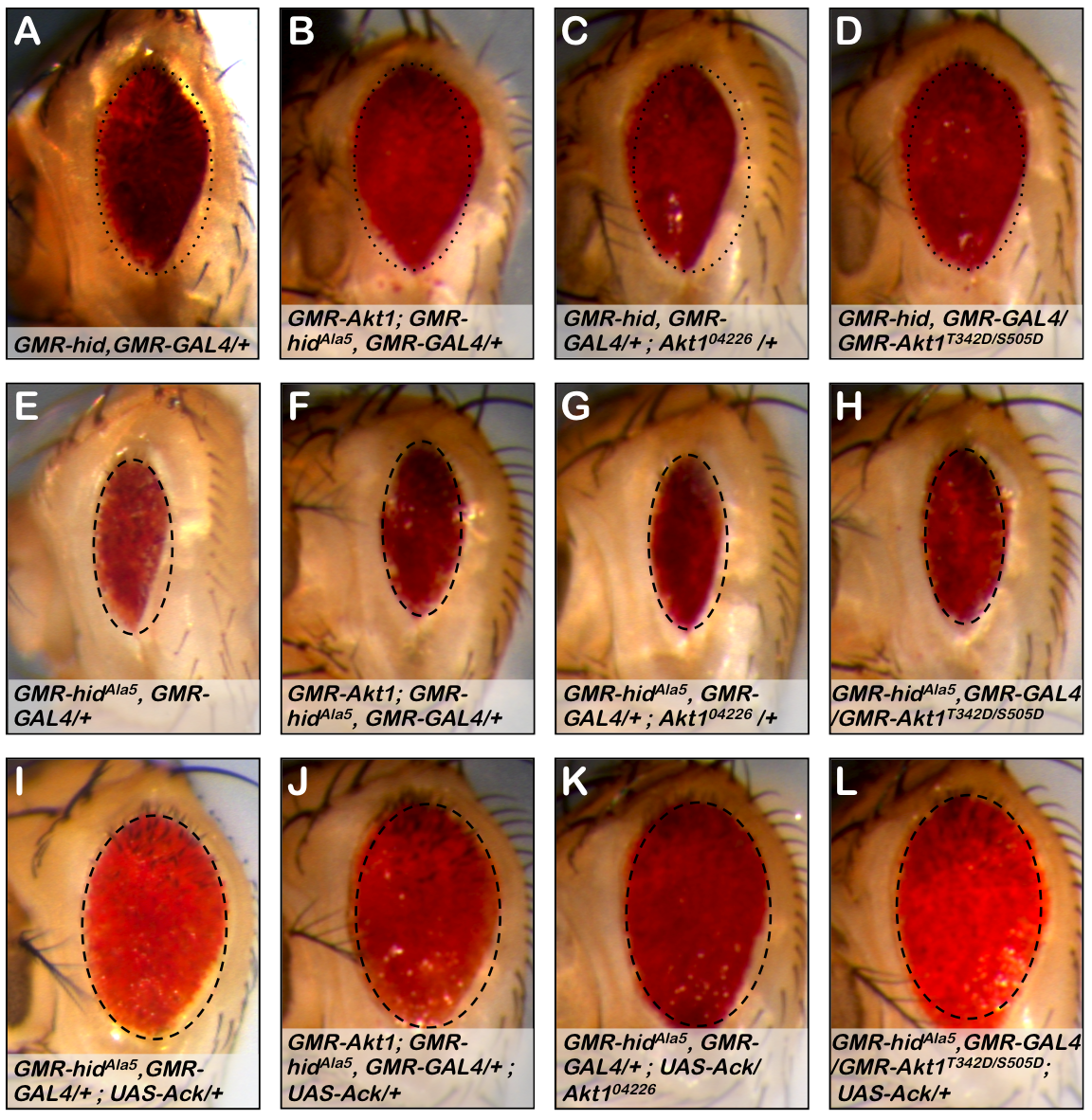

Supplement: Figure S3 — Akt1 does not modify the hid small eye phenotype. The eye size assay was used to assess the ability of Akt1 loss and gain of function to modify hid induced programmed cell death. Genotypes are indicated in each panel. To aid in eye size comparisons, the dotted oval in panel A is reproduced in panels B–D, the dashed oval in panel E is reproduced in panels F–H and the dashed oval in panel I is reproduced in panels J–L. (A–D) The GMR-hid, GMR-GAL4 genetic background combined with (B) Akt1 overexpression, (C) introduction of a loss of function allele Akt104226 or (D) overexpression of the constitutively activated mutant Akt1T342D/S505D. (E–H) The GMR-hidAla5, GMR-GAL4 genetic background combined with (F) Akt1 overexpression, (G) the loss of the function allele Akt104226 or (H) constitutively activated Akt1T342D/S505D overexpression. (I–L) Ack overexpression in the GMR-hidAla5, GMR-GAL4 genetic background combined with (J) Akt1 overexpression, (K) the loss of the function allele Akt104226 or (L) constitutively activated Akt1T342D/S505D overexpression. (TIF) [file pgen.1002725.s003.tif]

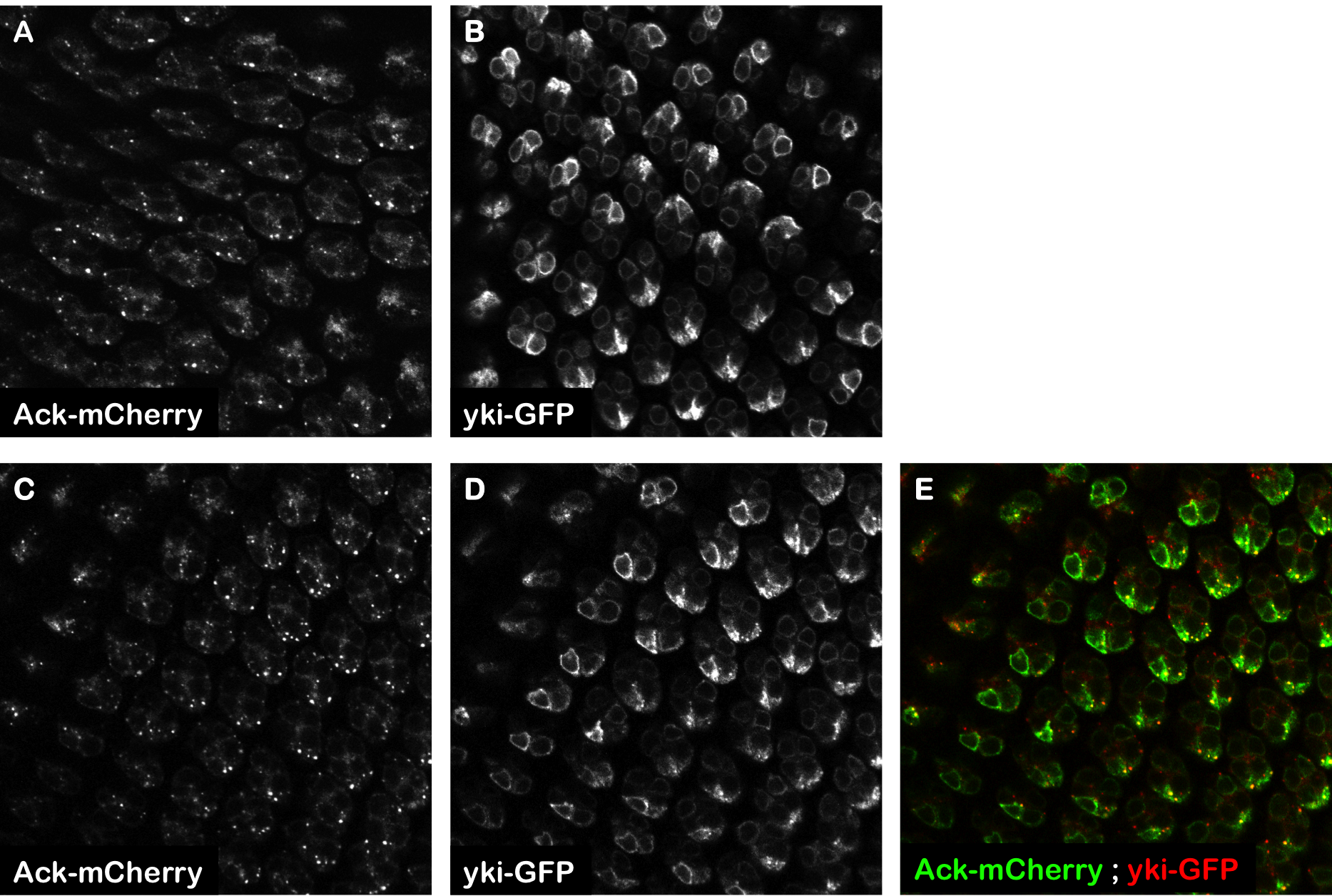

Supplement: Figure S4 — Ack-mCherry and yki-GFP subcellular localization in R-cells. Single plane confocal images show the subcellular localization of Ack-mCherry and yki-GFP expressed individually (A and B) or simultaneously (C–E) in third instar R-cells. Nuclei appear as open ringed structures in all panels. (A) Ack-mCherry is nuclear excluded and is found in the cytoplasm and in numerous cytoplasmic puncta. (B) Yki-GFP is also largely nuclear excluded and more diffusely distributed throughout the cytoplasm compared to Ack. (C–E) Simultaneous expression of Ack-mCherry (C) and yki-GFP (D) does not lead to increased nuclear localization of either protein, but does induce yki to co-localize into puncta with Ack. (E) An overlay of panels C and D showing Ack-mCherry (red) and yki-GFP (green) localization patterns. (TIF) [file pgen.1002725.s004.tif]

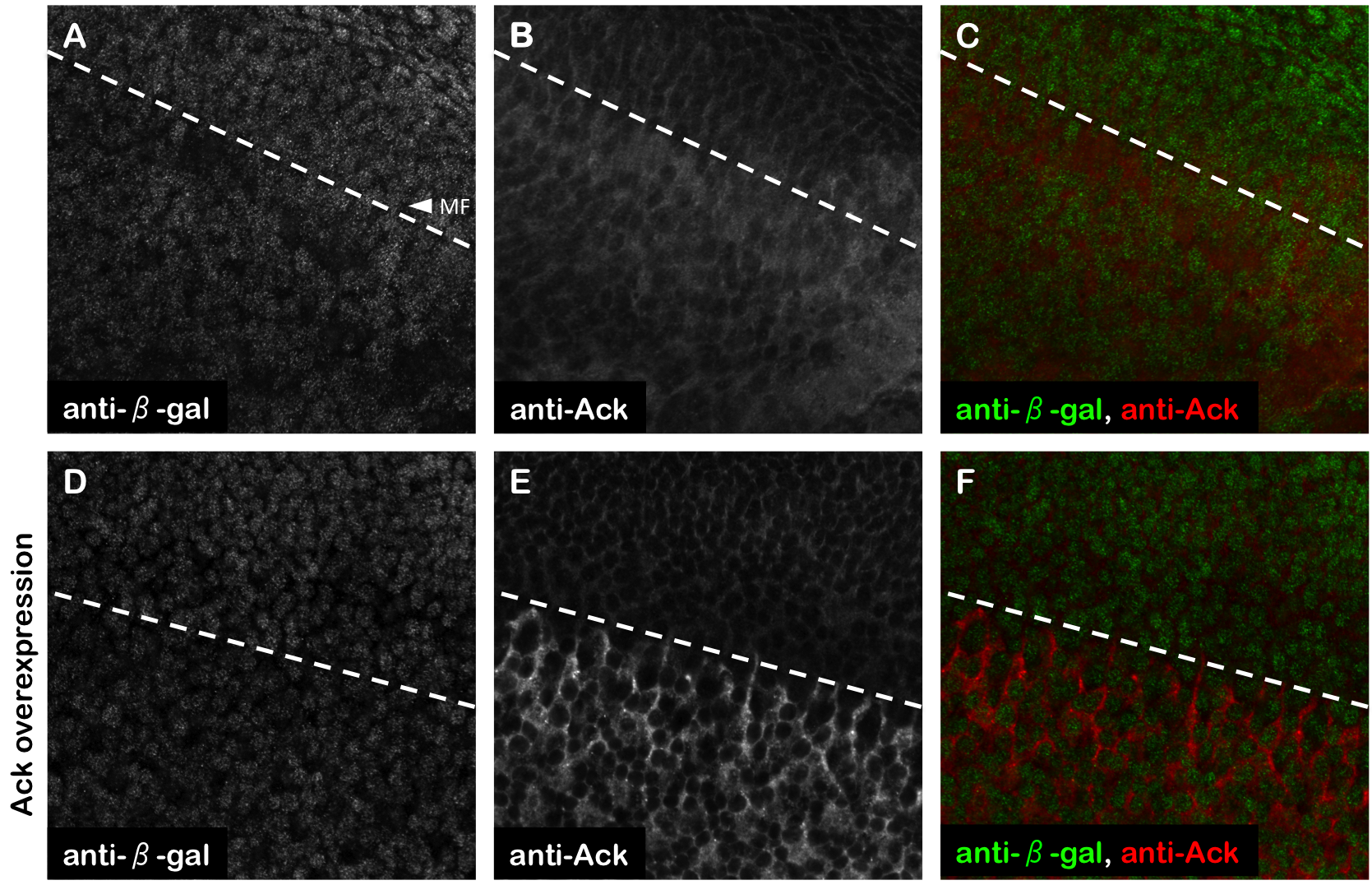

Supplement: Figure S5 — Ack expression does not induce transcription of yki targets. Confocal images of third instar eye discs analyzed for expression of Ack and beta-galactosidase driven by the ex-lacZ enhancer trap line ex697 in the absence (A–C) or presence (D–F) of Ack overexpression (posterior is down). The dashed line indicates the position of the morphogenetic furrow (MF). (A) Beta-galactosidase expression pattern in the absence of Ack overexpression shows similar levels of labeling posterior and anterior of the MF. (B) Ack expression is higher posterior of the MF. (C) An overlay of panels A and B showing beta-galactosidase (green) and Ack (red) expression. (D) Beta-galactosidase expression pattern in the presence of Ack overexpression again shows similar levels of labeling posterior and anterior of the MF. (E) Overexpression of Ack can be seen posterior to the MF due to GMR driven expression. (F) An overlay of panels D and E showing beta-galactosidase (green) and Ack (red) expression. (TIF) [file pgen.1002725.s005.tif]

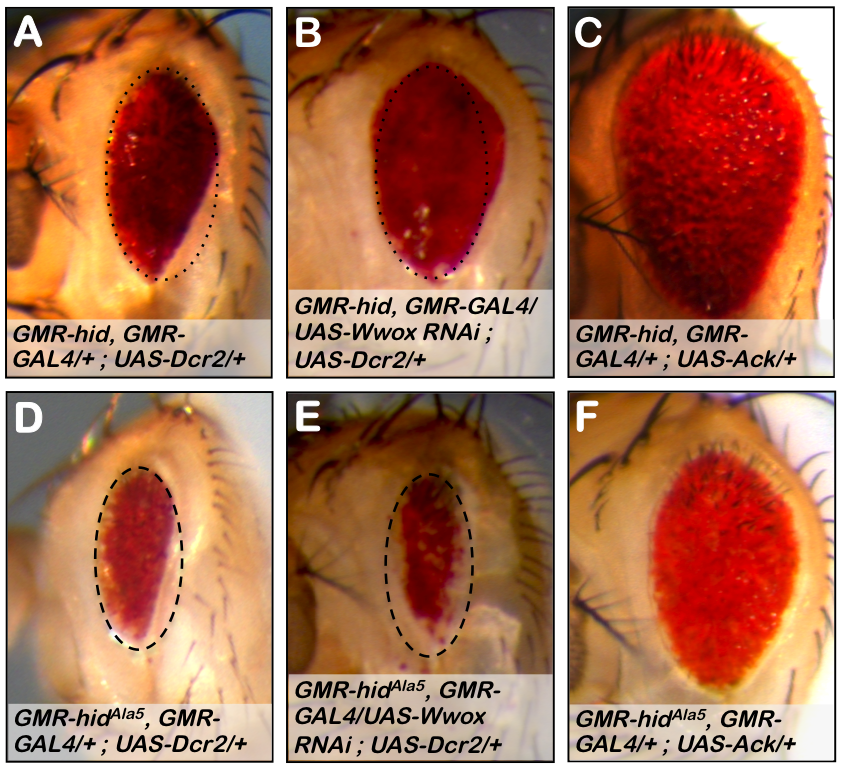

Supplement: Figure S6 — Suppression of hid induced small eye phenotypes by RNAi mediated knockdown of Wwox. The eye size assay was used to assess the ability of Wwox knockdown modify both hid and hidAla5 induced programmed cell death. (A) Hid expression in a GMR-GAL4 and UAS-Dcr2 expressing background produces a small eye phenotype. (B) RNAi mediated knockdown of Wwox suppresses the small eye phenotype. A dotted oval is used to aid in comparison. (C) Ack expression produces a larger increase in eye size in a similar genetic background. (D) HidAla5 expression in a GMR-GAL4 and UAS-Dcr2 expressing background also produces a small eye phenotype. (E) RNAi mediated knockdown of Wwox fails to modify the eye size in the GMR-hidAla5, GMR-GAL4; UAS-Dcr2 background. A dashed oval is drawn for comparison. (F) Ack expression suppresses the small phenotype induced in a GMR-hidAla5, GMR-GAL4 background. (TIF) [file pgen.1002725.s006.tif]
